# Supplementary material for: Integrating single-cell and single-nucleus datasets improves bulk RNA-seq deconvolution
Source: Cell Rep Methods. 2026 Mar 26;6(4):101346. doi: 10.1016/j.crmeth.2026.101346 (PMC13106970; doi:10.1016/j.crmeth.2026.101346)
Supplement: Document S1. Figures S1–S3 and Table S1 [file mmc1.pdf]

**Cell Reports Methods, Volume *6***

## Supplemental information

# Integrating single-cell and single-nucleus datasets improves bulk RNA-seq deconvolution

**Adriana Ivich and Casey S. Greene**

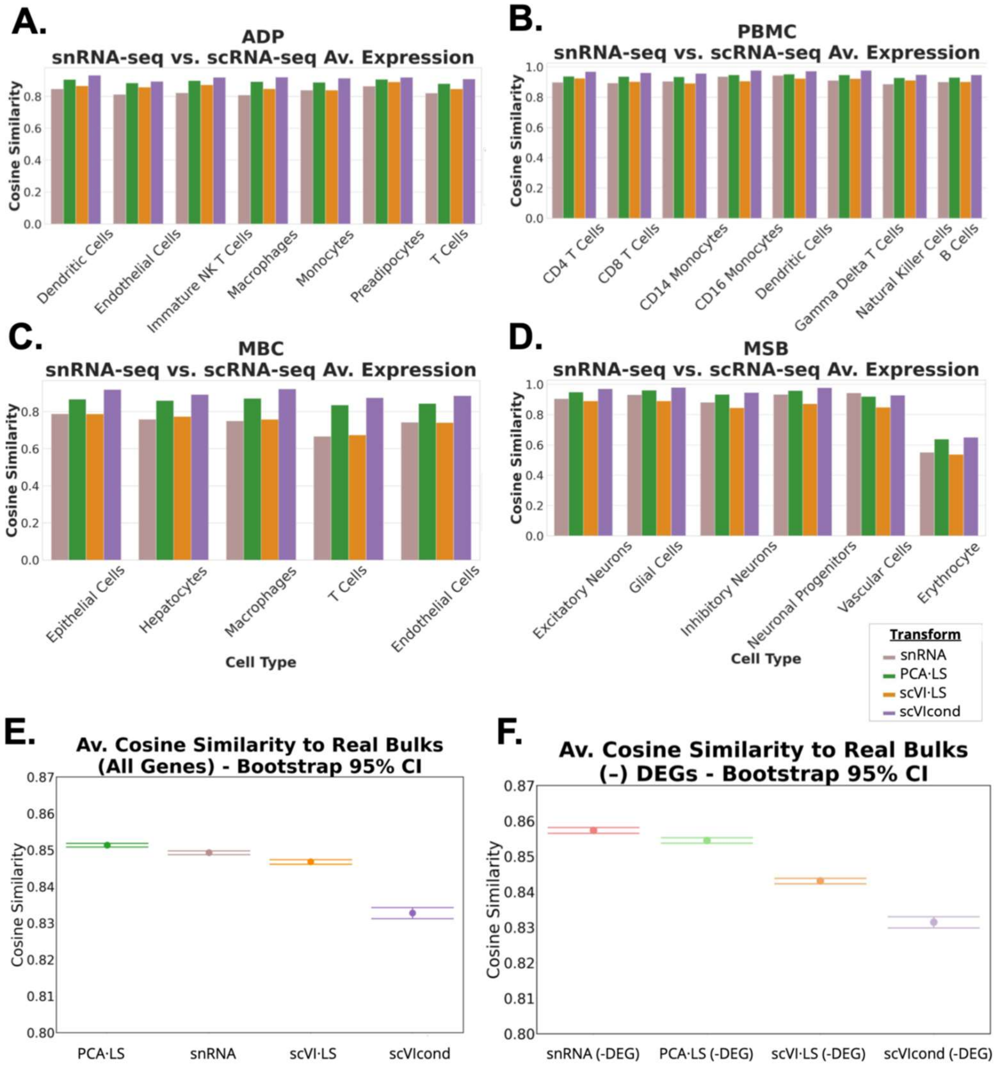


**Figure S1. ScVIcond improves cell-type alignment, whereas PCA·LS-transformed snRNA-seq best matches bulk expression, related to Figures 2–4.**

Comparison of transformed snRNA-seq data with matched scRNA-seq profiles and with bulk RNA-seq. (A–D) For each dataset (A: ADP, B: PBMC, C: MBC, D: MSB), we computed the cosine similarity between the mean CPM-normalized, log-transformed expression vector for each scRNA-seq cell type and the corresponding snRNA-seq cell type after transformation (PCA·LS, scVI·LS, scVIcond) or without transformation (raw snRNA-seq). Across datasets and cell types, transformations generally increase similarity relative to raw snRNA-seq; scVIcond shows the highest cell-type alignment overall with PCA·LS typically close behind, whereas scVI·LS is often comparable to raw snRNA-seq and can be lower for some MSB cell types. Cosine similarity captures the agreement in relative expression patterns (vector "shape") independent of overall amplitude (1 = identical patterns). (E) Adipose pseudobulks were created by aggregating scRNA-seq cell types and adding two snRNA-only cell types (adipocytes and neutrophils) either as raw snRNA-seq, transformed snRNA-seq, or using all snRNA-seq as a control; each pseudobulk was compared to 434 bulk RNA-seq profiles using cosine similarity in gene space. In this bulk comparison, pseudobulks with PCA·LS-transformed additions show higher similarity than pseudobulks with raw snRNA-seq additions, whereas scVIcond yields the lowest similarity. (F) Repeat of E using a different feature space in which genes differentially expressed between matched scRNA-seq and snRNA-seq cell types were removed (-DEG; see STAR Methods for DEG definitions). In the -DEG setting, DEG removal alone yields the highest bulk similarity, followed by PCA·LS−DEG. Dots indicate mean cosine similarity; bars show bootstrapped 95% confidence intervals; y-axes are truncated to highlight variation.

scRNA, single-cell RNA-seq; snRNA, single-nucleus RNA-seq; ADP, adipose tissue; PBMC, peripheral blood mononuclear cells; MBC, metastatic breast cancer; MSB, mouse E18 brain.

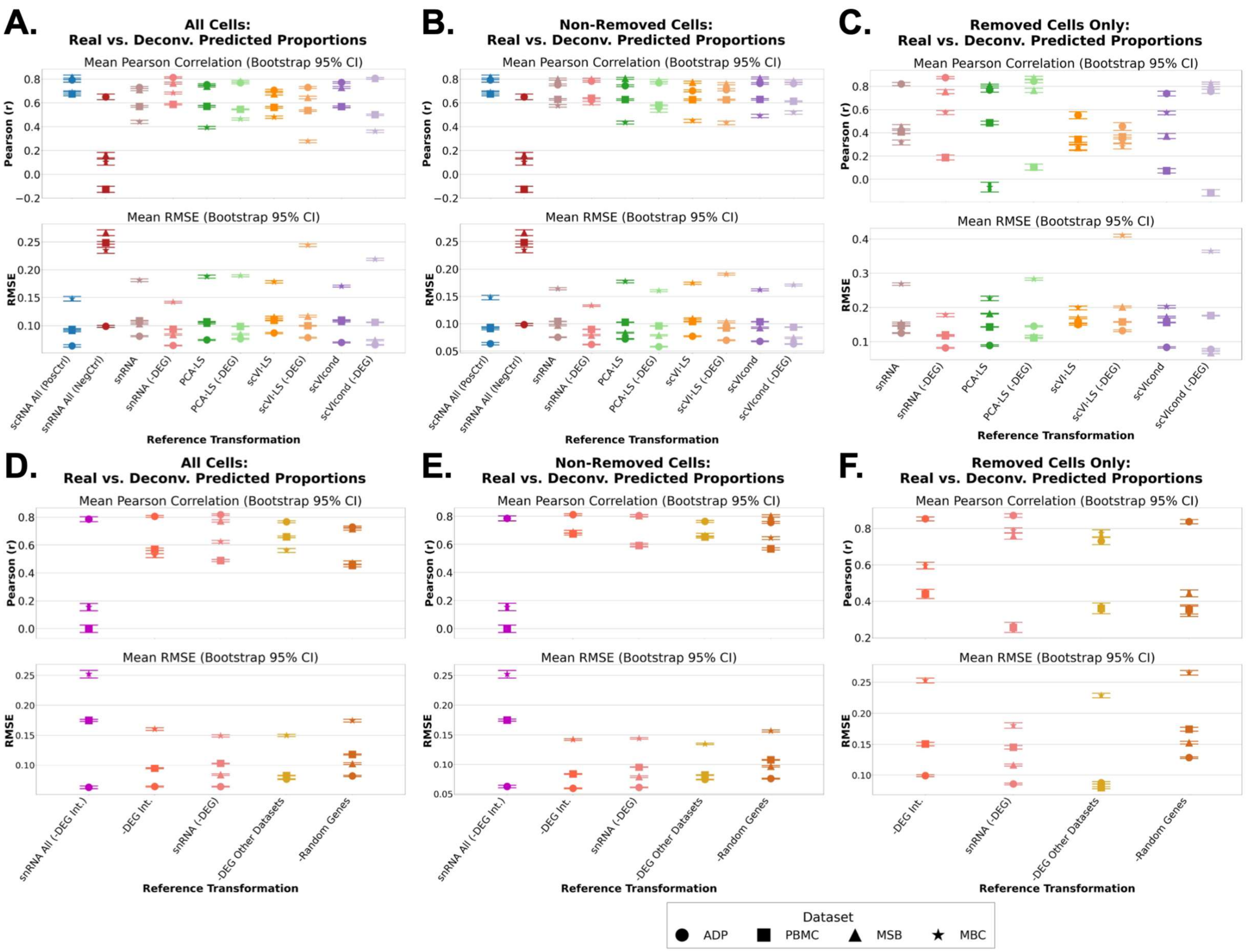


**Figure S2. Pseudobulk deconvolution accuracy with each cell type in scRNA-seq held out and transformed, related to Figure 2.**

Each plot shows the Pearson correlation value (top panels) and the RMSE values (bottom panel) for the ground truth pseudobulk (simulated) proportions and the predicted proportions. We hold out one cell type at a time from each dataset and replace that cell type's expression with a snRNA-seq equivalent with each of the transformations or controls (scRNA-seq All and snRNA-seq All) on the x axis of each plot. Each dot represents the mean metric (r or RMSE) across datasets (Figure 1B) and the bars represent the 95% bootstrapped confidence interval of the mean. We evaluated 3 scenarios (see Methods for details). (A and D) All cells included in performance metrics calculations. (D and E) Non-Removed cells only included in performance metrics calculations. (C and F) Only removed cells included in performance metrics calculations. Y-axes are truncated to highlight the variation. Combined metrics for all datasets are shown in Figure 2.

scRNA-seq: single-cell RNA-seq, RMSE: root mean squared error, snRNA-seq: single-nucleus RNA-seq.

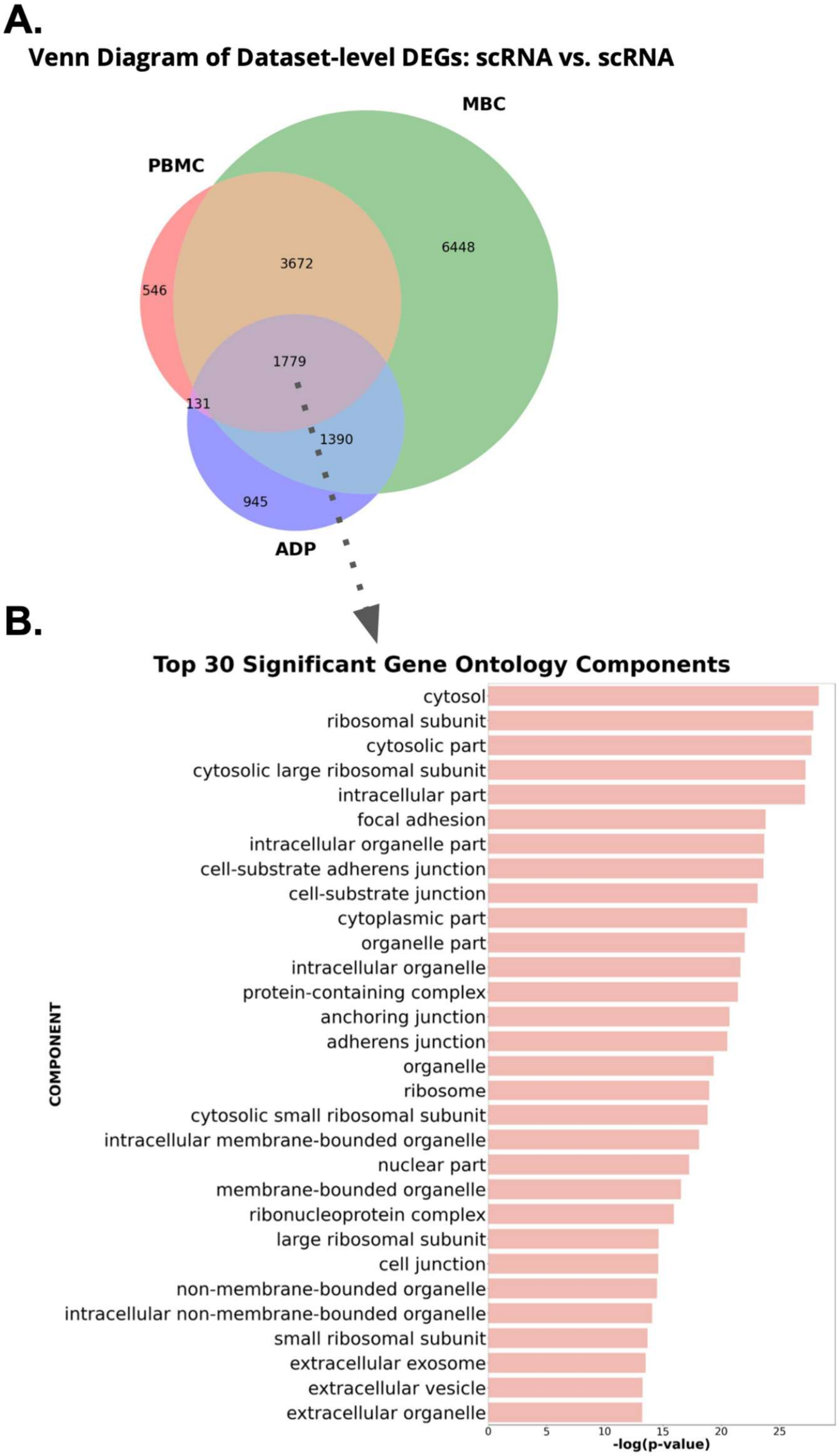


**Figure S3. Shared and unique scRNA-seq vs snRNA-seq differentially expressed genes across three human datasets, with GO-component enrichment of the common signature, related to Figures 2-4 and STAR Methods.**

DEGs between scRNA-seq and snRNA-seq cells of the same cell types in the three human datasets. a. Venn diagram showing the number of DEGs in common and distinct between cell types. b. Significant (GOrilla default of p <0.005) gene ontology terms (component) identified from the intersection genes (target) compared to the full gene list (all genes in common between datasets).

DEG: differentially expressed gene. ScRNA-seq: single-cell RNA-seq. snRNA-sequencing: single-nucleus RNA-sequencing. ADP: adipose tissue. PBMC: peripheral blood mononuclear cells. MBC: metastatic breast cancer.

**Differential Gene Expression per Dataset and Cell Type**
**(snRNA-seq vs. scRNA-seq)**

| Cell Type | Number of DEGs | Percentage of Total |
|---|---|---|
| **Peripheral Blood Mononuclear Cells (PBMC) Dataset** | | |
| Natural Killer Cells | 1915 | 8.73% |
| Gamma Delta T Cells | 2327 | 10.61% |
| Dendritic Cells | 1429 | 6.51% |
| CD4 T Cells | 4009 | 18.28% |
| CD8 T Cells | 3531 | 16.10% |
| B Cells | 3233 | 14.74% |
| CD16 Monocytes | 3785 | 9.61% |
| CD14 Monocytes | 3233 | 17.26% |
| **All Cell Types' Union** | **6128** | **27.94%** |
| **All Cell Types' Intersection** | **710** | **3.24%** |
| **Adipose Tissue (ADP) Dataset** | | |
| Preadipocytes | 3556 | 12.64% |
| T Cells | 520 | 1.85% |
| Immature NK T Cells | 336 | 1.19% |
| Macrophages | 1403 | 4.99% |
| **All Cell Types' Union** | **4245** | **15.09%** |
| **All Cell Types' Intersection** | **186** | **0.66%** |
| **Metastatic Breast Cancer (MBC) Dataset** | | |
| Endothelial Cells | 5422 | 17.88% |
| T Cells | 8738 | 28.82% |
| Immature NK T Cells | 8546 | 28.19% |
| Macrophages | 10081 | 33.25% |
| **All Cell Types' Union** | **13289** | **43.83%** |
| **All Cell Types' Intersection** | **3576** | **11.80%** |
| **Mouse E18 Brain (MSB) Dataset** | | |
| Inhibitory Neurons | 5069 | 19.61% |
| Neuronal Progenitors | 4299 | 16.63% |
| Glial Cells | 4455 | 17.24% |
| Excitatory Neurons | 5648 | 21.85%% |
| **All Cell Types' Union** | **7289** | **28.20%** |
| **All Cell Types' Intersection** | **2917** | **11.29%** |
| **Common Cells Between Datasets** | | |
| Macrophages (ADP and MBC) **Union** | 10531 | 3.9% |
| Macrophages (ADP and MBC) **Intersection** | 953 | 4.0% ADP, 66.7% MBC |
| T Cells (ADP and MBC) **Union** | 8911 | 9.06% |
| T Cells (ADP and MBC) **Intersection** | 347 | 9.45% ADP, 67.9% MBC |

**Table S1. Number of union and intersection genes per cell type in each of the datasets, related to Figures 2-4.** First row (left) shows the cell type, middle row shows the number of DEGs between that cell type in scRNA-seq vs. scRNA-seq. The right-most row shoes the percentage of total genes that are classified as DEGs. For each dataset, we note the number of genes that are in common between the cell types, both union (all) and intersection (common between all cell types). The human datasets contained 2 cell types in common across datasets, and we note the union and intersection of the DEGs between datasets as well.
DEG: differentially expressed gene. scRNA-seq: single-cell RNA-seq. snRNA-seq: single-nucleus RNA-seq.
