# Supplementary material for: Integrating single-cell and single-nucleus datasets improves bulk RNA-seq deconvolution
Source: Cell Rep Methods. 2026 Mar 26;6(4):101346. doi: 10.1016/j.crmeth.2026.101346 (PMC13106970; doi:10.1016/j.crmeth.2026.101346)
Supplement: Document S2. Article plus supplemental information [file mmc2.pdf]

Cell Reports
Methods

Article

Integrating single-cell and single-nucleus datasets improves bulk RNA-seq deconvolution

Graphical abstract

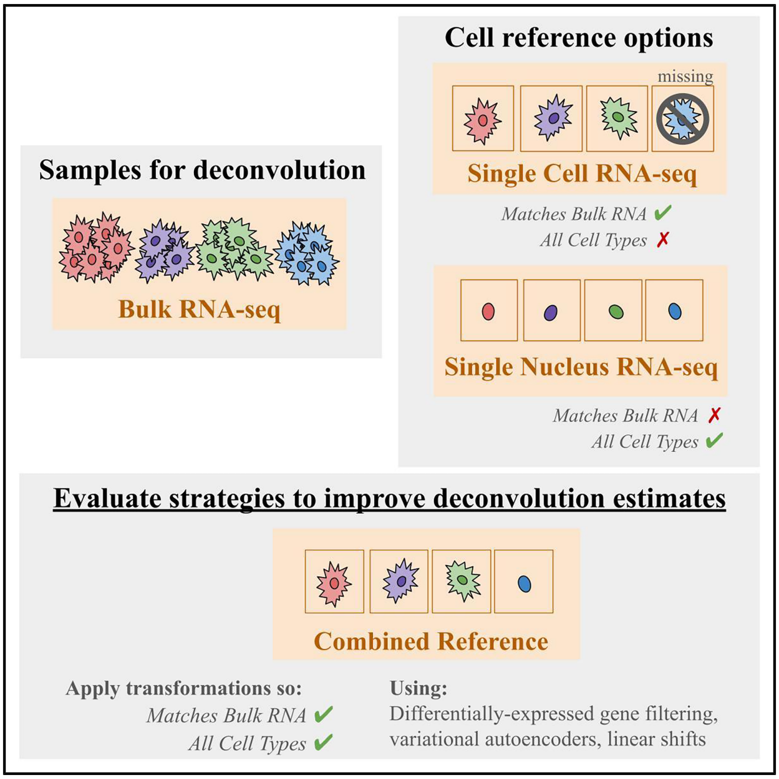

Highlights

- Reference modality choice impacts bulk RNA sequencing deconvolution accuracy
- Single-cell and single-nucleus references show tissue-dependent expression biases
- Modality-aware integration recovers accuracy comparable to single-cell references

Authors

Adriana Ivich, Casey S. Greene

Correspondence

casey.s.greene@cuanschutz.edu

In brief

Ivich and Greene evaluate how reference modality choice influences bulk RNA sequencing deconvolution accuracy. By comparing single-cell and single-nucleus references across tissues, they show that modality-aware strategies enable effective integration and provide practical guidance for reference selection in deconvolution analyses.

Ivich & Greene, 2026, Cell Reports Methods *6*, 101346
April 20, 2026 © 2026 The Authors. Published by Elsevier Inc.
https://doi.org/10.1016/j.crmeth.2026.101346

CellPress

Cell Reports Methods

CellPress
OPEN ACCESS

Article

Integrating single-cell and single-nucleus datasets improves bulk RNA-seq deconvolution

Adriana Ivich[1] and Casey S. Greene[1,2,*]
[1]Department of Biomedical Informatics, University of Colorado Anschutz, Aurora, CO 80045, USA
[2]Lead contact
*Correspondence: casey.s.greene@cuanschutz.edu
https://doi.org/10.1016/j.crmeth.2026.101346

MOTIVATION Reference-based bulk RNA-seq deconvolution relies on cell type expression profiles that accurately represent the cellular composition of bulk samples. Single-cell RNA sequencing (scRNA-seq or scRNA) provides whole-cell expression but often misses cell types due to dissociation-related loss. With single-nucleus RNA sequencing (snRNA-seq or snRNA) recovers, these cell populations can be sequenced but only include nuclear RNA. It remains unclear how these modality differences affect deconvolution accuracy, or whether scRNA-seq and snRNA-seq references can be combined without introducing error. This study was motivated to systematically assess the impact of each modality and to identify practical strategies for integrating scRNA-seq and snRNA-seq references for accurate and robust bulk deconvolution.

SUMMARY

Bulk RNA sequencing (RNA-seq) deconvolution typically uses single-cell RNA sequencing (scRNA-seq) references, but some cells are only detectable through single-nucleus RNA sequencing (snRNA-seq). Because snRNA-seq captures nuclear, not cytoplasmic, transcripts, its direct use as a reference could reduce deconvolution accuracy. We benchmarked integration strategies across four tissues, comparing principal component (PC)-based latent shifts, conditional and non-conditional scVI (single cell variational inference), and cross-modality differentially expressed gene (DEG) filtering. All approaches improved over raw snRNA-seq, but pruning cross-modality DEGs produced the largest gains, often matching or exceeding scRNA-only references. Conditional scVI performed comparably and was effective when matched scRNA-snRNA cell types were unavailable. In real adipose bulk samples, DEG pruning and conditional scVI provided the most robust cell-fraction estimates across donors and transformations. These results demonstrate that scRNA-seq should be prioritized as a reference when available, and we recommend appending snRNA-seq only after removing cross-modality DEGs; when DEG information is limited, conditional scVI is a practical alternative.

INTRODUCTION

Gene expression is typically studied using bulk RNA sequencing (bulk or bulk RNA-seq) of a variety of tissues. Bulk RNA-seq is a relatively cost-effective way to understand the transcriptional landscape of samples in the context of cancer, cell development, infectious disease, drug response prediction, etc.[1] Bulk RNA-seq can also be readily performed on frozen and formalin-fixed paraffin-embedded (FFPE) samples, expanding the accessibility and scope of bulk data.[1,2] Because bulk is widely available for many tissues, it can also be used to train deep learning models that require vast amounts of training data.[3–5]

However, bulk RNA-seq provides only indirect information about the cellular composition of the samples, limiting our understanding of the cell-type-specific contributions to the observed transcriptome.[6] The cell type proportions in a sample can reveal key details that influence the development of therapeutics, our understanding of the tumor microenvironment (TME), and more.[7,8] Single-cell RNA sequencing (scRNA-seq) provides gene expression measurements of individual cells. Each individual whole cell is isolated or barcoded, and the transcriptome is sequenced.[6] ScRNA-seq gene expression counts contain both cytoplasmic and nuclear RNA and, in an ideal world, produce data directly comparable to bulk RNA-seq.

Bulk deconvolution enables researchers to estimate cell type proportions and cell-type-specific expression from vast archival bulk samples.[9–11] Bulk deconvolution describes the process of estimating the proportions of discrete cell types in a bulk transcriptomic sample. Accurately deconvolving bulk data could reveal changes in the TME, transcriptomic heterogeneity in

© 2026 The Authors. Published by Elsevier Inc.
This is an open access article under the CC BY license (http://creativecommons.org/licenses/by/4.0/).

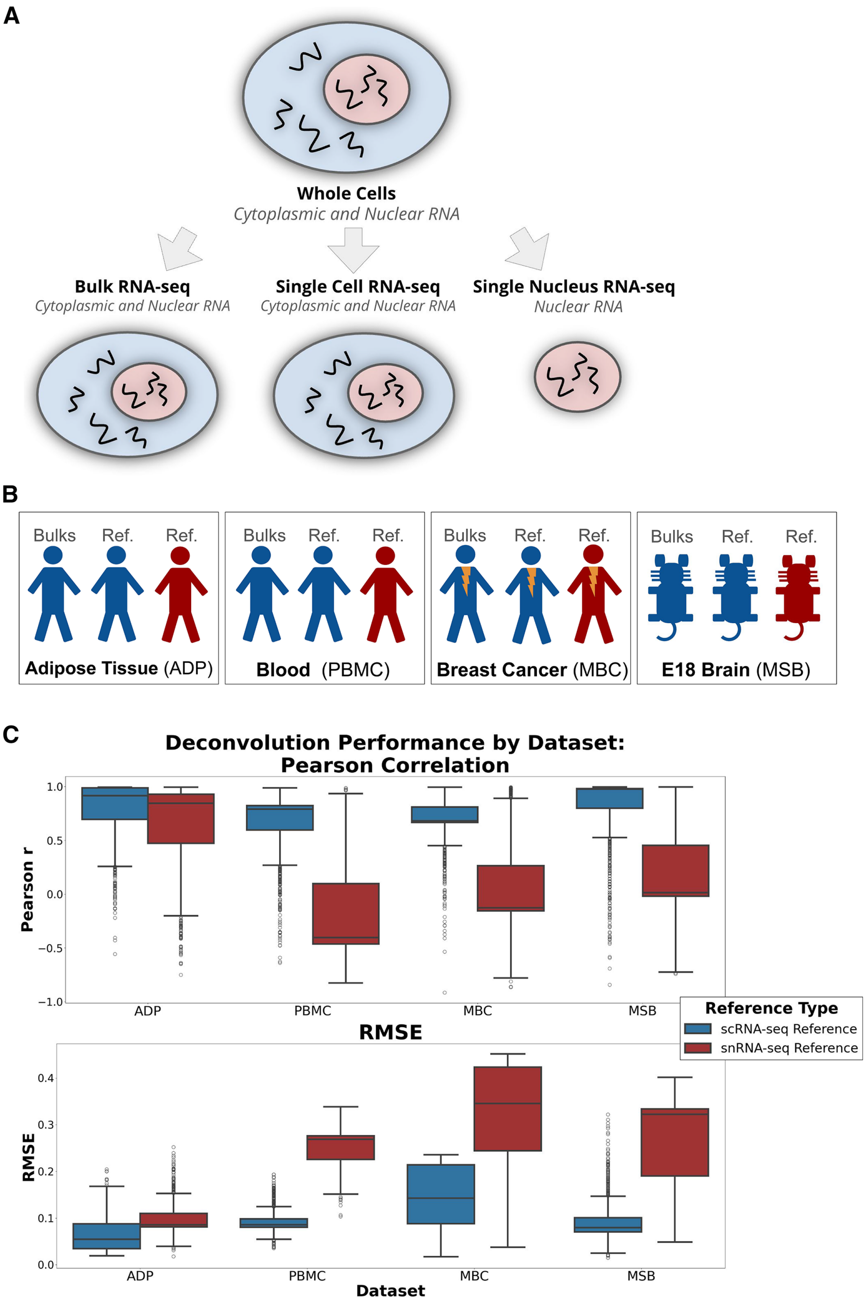

Figure 1. Schematics of experimental motivation and datasets used

(A) RNA sequencing yields different datasets depending on cell proportion that is sequenced. Bulk RNA-seq and scRNA-seq) contain counts of both cytoplasmic and nuclear RNA. However, single-nucleus RNA-seq contains only nuclear RNA portion.

(B) Our study uses four RNA-seq dataset types: adipose tissue (ADP), peripheral blood mononuclear cells (PBMC), metastatic breast cancer (MBC), and developmental mouse brain (MSB). These datasets encompass 4 distinct and varied situations of cell loss across species.

(C) Comparison of deconvolution performance (Pearson and RMSE) of pseudobulks made from scRNA-seq using an scRNA-seq reference and an snRNA-seq reference with same cell types. Boxplots show the distribution of values across pseudobulk samples; center line indicates the median, boxes represent the interquartile range, and whiskers indicate the range of values within the 1.5× the interquartile range.

RMSE, root mean squared error; scRNA-seq, single-cell RNA-seq; snRNA-seq, single-nucleus RNA-seq.

response to treatments, and other key factors that could influence survival.

Reference-based deconvolution methods generally assume a strong concordance between the gene expression observed in the cell references used and the bulk at hand, so researchers often use an scRNA-seq dataset as a reference for cell type expression.[12] However, this assumption is compromised in cases where there are expression changes and notable cell losses due to the dissociation procedures or microfluidic devices used in many scRNA-seq protocols. Apart from dissociation-specific marker upregulation,[12] scRNA-seq protocols can cause cell types to be lost.[13] When cell types present in bulk are missing from single-cell observations, they substantially reduce deconvolution performance,[13] making cell proportion estimates unreliable. In these cases, researchers often use single-nucleus RNA sequencing (snRNA-seq) as a reference[14,15] or combine both modalities.[16,17] Cells that are sensitive or difficult to dissociate are still often observable with snRNA-seq,[18–20] and cell types not often found with scRNA-seq can be seen in snRNA-seq data.[20] However, snRNA-seq captures nuclear RNA (Figure 1A), and the loss of cytoplasmic RNA compromises the assumption of high concordance.

Previous work has focused on deconvolution methods that expect or can use snRNA-seq references exclusively within certain difficult-to-assay tissues.[21–23] Other work has focused on aligning the bulk expression with the reference expression, which mitigates the gap between cytoplasmic and nuclear RNA present in snRNA-seq datasets, such as the deconvolution methods *SQUID*[24] and *BISQUE*.[23] These methods use snRNA-seq references but rely either on matched tissue (bulk and reference from the same sample) or cell proportions in the reference to match the bulk to fit the bulk expression using observed cell proportions in the reference. Both are unfeasible when the cell type references available are from different datasets or require distinct modalities to include all cell types, i.e., with unmatched snRNA-seq samples and scRNA-seq data that do not contain

CellPress
OPEN ACCESS

all cell types. Also, the constraint that bulk cell type proportions and single-nucleus proportions must match is challenging to confirm, as many tissue types can be heterogeneous in composition. While the distinction between snRNA-seq and scRNA-seq references is recognized and clearly influences deconvolution, the impact of simple transformations applied to mixed references on deconvolution performance using methods that rely on scRNA-seq is underexamined. The present study aims to investigate whether this mixed-modality reference can improve deconvolution performance in a benchmarked method that expects scRNA-seq as a reference.

In this study, we compare deconvolution performance in the context of simple transformations applied to scRNA-seq vs. snRNA-seq references. We assess gene filtering, linear principal-component analysis (PCA) neighbor-based shifts, and non-linear scVI-based transformations.[25,26] We benchmarked transformations on four datasets: human adipose tissue, blood, metastatic breast-cancer liver tissue, and embryonic mouse brain. We systematically substituted one scRNA-derived cell type with its (untransformed or transformed) snRNA-seq equivalent and evaluated concordance against pseudobulks and real bulk samples and robustness across patients. We focus our study on BayesPrism deconvolution and test the transformation's generalization using other deconvolution methods. While using snRNA-seq references alone substantially reduced deconvolution accuracy, simple transformations often closed the gap. In addition, filtering genes that were consistently differentially expressed between scRNA-seq and snRNA-seq cell types across tissues often improved performance in unrelated datasets to a level consistent with other transformations.

RESULTS

Pseudobulk deconvolution with scRNA-seq and snRNA-seq established baseline performance

Our goal was to establish an experimental framework to compare scRNA-seq and snRNA-seq references under the assumption that snRNA-seq would represent a complete set of cell types with incomplete RNA (only nuclear), while scRNA-seq would represent the complete expression measurements for cells but for an incomplete set of types. To simulate this setting, we created pseudobulks with known proportions from one scRNA-seq sample—meaning one batch or dataset from one single patient (independent to the other datasets). We then deconvolved all pseudobulks with either an scRNA-seq or snRNA-seq reference derived from a different sample (i.e., one batch) of the same tissue type containing the same cell types in equal numbers. We assessed performance using root mean squared error (RMSE) and Pearson correlation of cell type proportions (see STAR Methods for more details). We repeated this in four datasets: adipose tissue (ADP), peripheral blood mononuclear cells (PBMCs), metastatic breast cancer (MBC), and one mouse brain (developmental) (MSB) (Figure 1B). Consistent with expectations, scRNA-seq yielded a higher Pearson correlation and a lower RMSE than the snRNA-seq reference across all datasets (Figure 1C).

We assessed the significance of differences using two-sample t tests of Pearson correlation and RMSE across both reference types within each dataset. In the adipose dataset, the scRNA-seq reference produced significantly higher Pearson correlations than the snRNA-seq reference ($t = 10.17$, $p < 1e-6$), while RMSE values were significantly lower for scRNA-seq ($t = -23.41$, $p < 1e-6$), indicating more accurate deconvolution.

Highly significant differences in deconvolution performance, i.e., RMSE and Pearson correlation, were observed across all datasets, with $p < 1e-6$. This poor performance of the of snRNA-seq references is consistent with the strong nuclear-specific expression biases in raw snRNA-seq (e.g., reduced cytoplasmic gene detection, increased intronic signal, and gene-length effects), which distort the relative expression patterns that BayesPrism relies on. Because BayesPrism models each reference profile as the expected generative distribution of the bulk mixture, these modality-specific discrepancies introduce systematic directionality errors that the model cannot correct.

Multiple approaches can integrate snRNA-seq and scRNA-seq references

We implemented a set of controls and transformations (Table 1) across a set of potential algorithmic selections. These were the source modality for each cell's expression, the operation type (cell type specific or global), the transformation applied (e.g., scVI conditional, latent space shifts, etc.), and the final gene set used in the references.

To first test whether our transformations make snRNA-seq profiles closer to their scRNA-seq and bulk (nuclear and cytoplasmic RNA as well) counterparts, we applied the three cell transformation strategies (scVI conditional [scVIcond], scVI and PCA latent space shifts [scVI LS and PCA LS]) plus DEG-filtered variants) to four datasets (details in STAR Methods). At the single-cell level, assessing with cosine-similarity across the four datasets revealed that transforming each snRNA-seq cell type with the scVIcond model most closely aligned it to the matching scRNA-seq signature, with PCA LS a close second. In this context, the latent-shift adjustment offered little or no gain (Figures S1A–S1D). Notably, erythrocytes showed the lowest cosine similarity, which we hypothesize is because mature erythrocytes do not have a nucleus or organelles and have little RNA left,[27] and previous work by our group has suggested that they are heavily lysed and lost in dissociation protocols.[28]

The high scVIcond cosine similarity between scRNA-seq and snRNA-seq-transformed cell types suggests that the difference in scRNA-seq vs. snRNA-seq expression is non-linear and well captured and changed in a conditional variational autoencoder (VAE) framework. This differs from the scVI LS transform that applies a global shift rather than a per-cell calculated shift and performs worse, suggesting that the difference is cell or cell type specific rather than global.

When we aggregated the transformed fat-cell and neutrophil profiles into 100 synthetic pseudobulks and compared them with real bulk RNA-seq, PCA LS-based pseudobulks best resembled bulk expression, while scVIcond-transformed pseudobulks were least similar; after removing cell-type-specific DEGs, the highest bulk-level similarity was achieved simply by using DEG-filtered snRNA-seq, followed by the PCA LS (-DEG) transform (Figures S1E and S1F).

These results suggest that PCA LS-based pseudobulks most closely resembled bulk because the PCA LS-neighbor approach

Table 1. Description of each control and transformation evaluated

Reference name	Genes filtered out	Cells types in reference	Transformation applied
scRNA All (PosCtlr)	none	all scRNA cell types	none (control)
snRNA All (NegCtlr)	none	all snRNA cell types	none (control)
snRNA All (-DEG Int.)	genes found to be DEG across all human datasets (intersection shown in Figure S3)	all snRNA cell types	none
snRNA	none	all scRNA cell types; added cell type(s) from snRNA	none, adding snRNA cell type as is
snRNA (-DEG)	genes DE between scRNA and snRNA in each cell type (excluding the snRNA cell type)	all scRNA cell types; Added cell type(s) from snRNA	none
PCA LS	none	all scRNA cell types; Added cell type(s) from snRNA	none to scRNA cell types; neighbor-based shift in the PC space to snRNA cell types
PCA LS (-DEG)	genes DE between scRNA and snRNA in each cell type (excluding the snRNA cell type)	all scRNA cell types; added cell type(s) from snRNA	none to scRNA cell types; neighbor-based shift in the PC space to snRNA cell types
scVI LS	none	all scRNA cell types; added cell type(s) from snRNA	none to scRNA cell types; neighbor-based shift in the VAE latent space to snRNA cell types
scVI LS (-DEG)	genes DE between scRNA and snRNA in each cell type (excluding the snRNA cell type(s))	all scRNA cell types; added cell type(s) from snRNA	none to scRNA cell types; neighbor-based shift in the VAE latent space to snRNA cell types
scVIcond	none	all scRNA cell types; added cell type(s) from snRNA	none to scRNA cell types; conditional VAE, label encoded and switched to snRNA cell types
scVIcond (-DEG)	genes DE between scRNA and snRNA in each cell type (excluding the snRNA cell type(s))	all scRNA cell types; added cell type(s) from snRNA	none to scRNA cell types; conditional VAE, label encoded and switched to snRNA cell types
-DEG Int.	genes found to be DEG across all human datasets (intersection shown in Figure S3), except genes DE in the snRNA cell type(s)	all scRNA cell types; added cell type(s) from snRNA	none
-DEG Other Datasets	genes found to be DEG in the other (human only) datasets	all scRNA cell types; added cell type(s) from snRNA	none
-Random Genes	random set of genes of equal number and excluding the DEGs	all scRNA cell types; added cell type(s) from snRNA	none

Reference name shows the colors used throughout the plots for each control and transformation. The table shows the reference name as is referred in the main text and figures, the origin of the cell's expression included in the reference, whether the transformation is applied to all cells or only one cell type (snRNA-seq), the transformation type, and the genes that are present in the final reference. "All" genes included in reference (right column) refers to all genes in common between the references and pseudobulks/bulks. Note that some transformations only contain one snRNA-seq cell type, and the rest of the cells' expression comes from scRNA-seq. The first two references listed (scRNA All [PosCtrl] and snRNA All [NegCtrl]) contain all cells from only one reference with no transformation applied as controls. The snRNA All (-DEG Int.) is the only other reference type that contains all cell types from one modality, snRNA-seq. scRNA-seq, single-cell RNA-seq; snRNA-seq, single-nucleus RNA-seq.

effectively substitutes the fully nuclear expression profile with the expression profile of the most similar scRNA cells. As a result, the aggregated signal becomes more reflective of whole-cell RNA, which bulk represents. In contrast, scVIcond maintains more fine-grained, cell-type-specific structure when aligning nuclei to scRNA. That subtle cellular structure is desirable for single-cell harmonization but becomes less bulk-like when aggregated, explaining why scVIcond performs best at the cell level but not after collapsing cells into pseudobulks. Removing snRNA-specific DEGs further improves concordance by eliminating genes systematically biased toward nuclear expression, allowing the remaining features to better resemble true whole-cell RNA content.

Transforming snRNA-seq measurements using an scRNA-seq reference improves deconvolution

We then evaluated the performance of deconvolution with ground truth proportions. For each tissue type (Figure 1B), we used 3 samples: one scRNA-seq to create pseudobulks, one scRNA-seq for reference, and one snRNA-seq used to apply

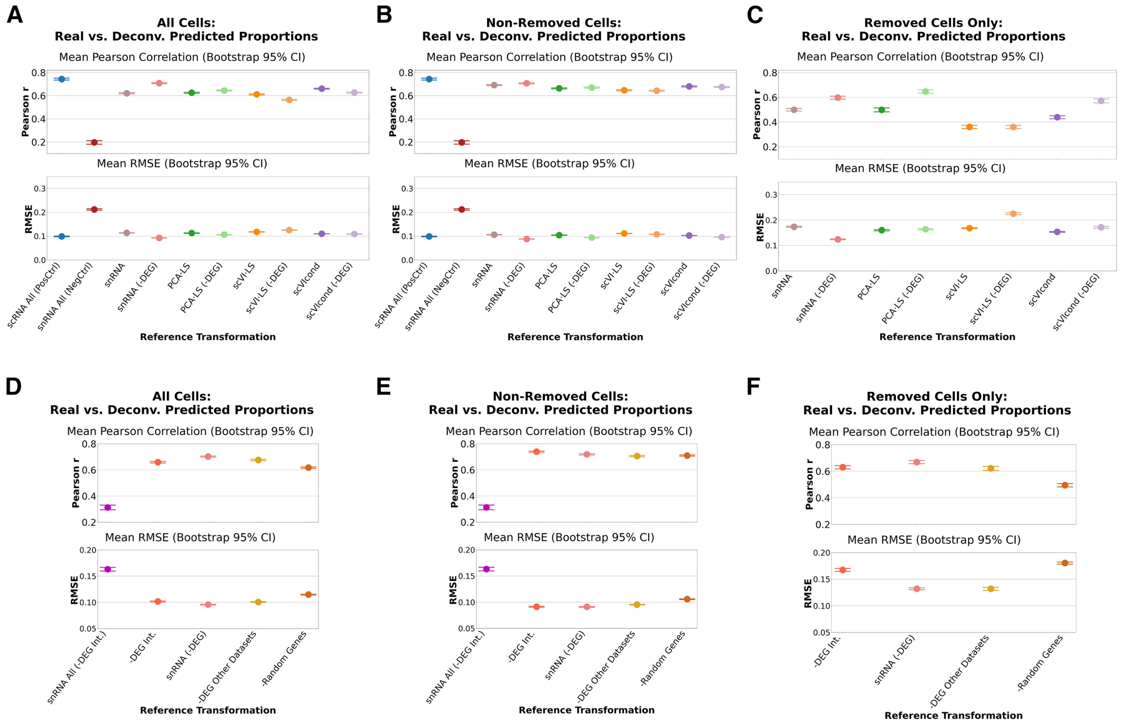

Figure 2. Pseudobulk deconvolution accuracy with each cell type in scRNA-seq held out and transformed

(A–F) Each plot shows the Pearson correlation value (top) and the RMSE values (bottom) for the ground truth pseudobulk (simulated) proportions and the predicted proportions. We hold out one cell type at a time from each scRNA-seq dataset and replace that cell type's expression with a snRNA-seq equivalent with each of the transformations or controls (scRNA All and snRNA All) on the *x* axis of each plot. Each dot represents the mean metric (correlation or RMSE) across datasets (Figure 1B), and the bars represent the 95% bootstrapped confidence interval of the mean. We evaluated 3 scenarios (see STAR Methods for details). Per dataset metrics can be seen in Figure S2. *y* axes are truncated to highlight the variation.

(A and D) All cells included in performance metrics calculations.

(B and E) Non removed cells only included in performance metrics calculations.

(C and F) Only removed cells included in performance metrics calculations.

scRNA-seq, single-cell RNA-seq; RMSE, root mean squared error; snRNA-seq/snRNA, single-nucleus RNA-seq.

and test our transformations. We created pseudobulks from the scRNA-seq-designated sample with all cell types with more than 50 cells (see STAR Methods for details of pseudobulk creation). We created two control references for deconvolution for each tissue type: one with all cells from the reference scRNA-seq sample (scRNA All [PosCtrl]) and a second with all cells from the snRNA-seq sample (snRNA All [NegCtrl]).

In each cell reference for deconvolution, we held out one cell type at a time from an scRNA-seq sample and replaced that cell's expression with an equivalent from the snRNA-seq sample, yielding a complete but mixed-modality reference. This snRNA-seq cell type was either added as is (snRNA) or with each of our transformations (PCA LS, scVIcond, scVI LS, and the same without DEGs; see Table 1 and STAR Methods for more details). All the transformations contain the same cell type's expression, just from a different data modality source. We hypothesized that the transformations would improve the deconvolution performance when compared to the snRNA All (NegCtrl) and would be closer in performance to the scRNA All (PosCtrl) reference. This positive control reflects the ideal but unexpected scenario where all cells available in the bulk are also present in scRNA-seq.

We evaluated the deconvolution in three different groups, each corresponding to the specific cell type proportions of interest. In the first group, "All Cells," we evaluated the performance in estimating the cell type proportions of all cell types in the pseudobulk, independent of held-out status. The second group, "Non-Removed Cells" evaluated performance only on the subset of cell types that were not removed, assessing the impact on cell types that were present in scRNA-seq data. Lastly, we subset performance to "Removed Cell Only" to examine the accuracy of proportion estimates for the cell types that were absent in the scRNA-seq data.

We grouped all performance metrics across the four tissue types (Figure 2: dot = mean; bars = 95% bootstrapped confidence interval [CI] of the mean, 1,000 iterations). For All Cells (Figure 2A), the scRNA All (PosCtrl) achieved the highest Pearson correlation, followed closely by the snRNA-DEG transformation and then scVIcond transformation. The remaining methods performed somewhat comparably, but much better than the snRNA All (NegCtrl). Using all cells from an snRNA-seq sample as reference yielded the worst performance in both RMSE and Pearson correlation metrics, with the upper 95% CI failing to exceed a correlation higher than 0.2. Surprisingly, the snRNA-DEG

transformation outperformed the positive control; in fact, the positive control's RMSE was comparable to that of PCA LS (-DEG). Removing DEGs likely eliminates precisely the genes whose modality-specific differences violate BayesPrism's modeling assumptions, effectively removing non-biological variation even from the scRNA-seq-only positive control. Notably, removal of DEGs boosted performance in all transformations (e.g., scVIcond-DEG achieved higher Pearson correlation and lower RMSE than scVIcond). This consistent improvement suggests that a substantial portion of the scRNA-snRNA mismatch arises from a relatively small set of modality-driven genes and that suppressing their contribution stabilizes the reference geometry for deconvolution.

Across tissue types, these trends held generally. The per-tissue deconvolution performance is shown in Figure S2. Notably, the negative control reference in the ADP dataset had surprisingly good performance (mean ≈0.65), although it dipped as low as −0.2 in other datasets. Nevertheless, it exhibited the lowest performance in all settings. This elevated performance in adipose tissue is consistent with reports that adipocytes show relatively modest nuclear-cytoplasmic expression differences compared to immune or neuronal cells,[29] making raw snRNA-seq a closer approximation of whole-cell profiles in this tissue. As a result, the modality mismatch is reduced but not eliminated, relative to the other datasets.

In the Non-Removed Cells scenario, the overall patterns persisted: the positive control again led in Pearson correlation, and the negative control correlation remained below 0.2 (Figure 2B). In RMSE, several DEG-removed methods (snRNA-DEG, PCA LS-DEG, and scVIcond-DEG) again outperformed the positive control, and the performances of scVIcond and scVI LS-DEG were comparable. Per-dataset analysis mirrored the All Cells results, with the ADP dataset showing particularly high Pearson correlation and low RMSE across methods compared to the other datasets yet still ranking the negative control worst within that sample (Figure S2B). This improvement reflects BayesPrism's joint inference: when the held-out cell type is poorly modeled, as in the negative control, its mis-specified profile perturbs the shared likelihood and degrades estimates for all other cell types. Removing modality-specific DEGs reduces this spillover by stabilizing the shared gene space, allowing cleaner separation of the remaining cell identities.

The Removed Cell Only scenario is unevaluable with the positive and negative controls, since those references did not include removed cells. In this scenario, we observed the largest inter-transform variability in the mean Pearson correlation and RMSE (Figure 2C), hinting at cell-type-specific advantages to each transform. This high transform-to-transform variability was also observed at the dataset level (Figure S2C). SnRNA-DEG achieved the highest Pearson correlation and lowest RMSE (Figure 2C). The PCA LS-DEG and scVIcond-DEG transformations yielded Pearson correlation values higher than the positive control, and all transformations (except scVI LS) achieved lower (or comparable for scVI LS-DEG) RMSE. At the per-dataset resolution, the scVIcond mean Pearson correlation was greatly decreased by the peripheral blood mononuclear cell (PBMC) dataset, and without considering this dataset, the scVIcond would outperform all other transform's Pearson correlations (Figure S2C). This could suggest that the conditional VAE model for the PBMC dataset might require different training parameters or datasets, likely because PBMC contains many closely related immune subsets for which conditional VAE models require more data or tuning to accurately learn modality shifts. The MSB dataset exhibited uniformly poorer RMSE across all transforms (Figure S2C), consistent with the stronger nuclear retention and developmental gradients in embryonic brain, which make nuclear-to-cytoplasmic alignment inherently more difficult.[30]

Together, these findings revealed that removing DEGs (-DEG) was sufficient to improve deconvolution performance across many scenarios and datasets. The DEG-pruned transformations, especially the snRNA-DEG, enhanced deconvolution accuracy across all evaluation scenarios, often in line with positive control and deep learning performances.

Selective pruning of dataset-specific and cross-dataset DEGs improves deconvolution accuracy

We then aimed to test whether removing DEGs observed between modalities across multiple datasets was sufficient to improve deconvolution performance. This would avoid the need to have matched snRNA-seq and scRNA-seq data from enough participants to have well-powered tests of differential expression. We considered that DEG between cell types observed in multiple other tissue datasets (human only) could enable the filtering of genes that translate across tissue types (-DEG Other Datasets). We also removed the intersection of genes classified as differentially expressed in all three human datasets in at least one cell type (-DEG Int.). This list of genes is described in Figure S3. Moreover, as a negative control, we tested removing a random set of genes matched in size to the -DEG set to evaluate whether simply removing genes or features improved performance. Lastly, to test whether gene pruning would be sufficient to obtain good performance for a full snRNA-seq reference, we also tested a full snRNA-seq reference without the DEGs found across all human datasets (-DEG Int.). All pruned gene lists and references created are summarized in Table 1. We evaluated the impact of these gene-removal strategies on deconvolution accuracy across the three evaluation scenarios (All Cells, Non-Removed Cells, and Removed Cells).

In the All Cells scenario, pruning the dataset-specific DEGs (the same as snRNA-DEG, as aforementioned) again delivered the best performance, achieving the highest mean Pearson correlation and the lowest RMSE (Figure 2D). This reflects the fact that dataset-specific DEGs capture the strongest modality-driven distortions for the specific cell types present in each dataset; removing these genes directly mitigates the mismatch that BayesPrism would otherwise misinterpret as biological divergence. By contrast, the pruned full snRNA-seq reference snRNA All (-DEG Int.) produced the poorest accuracy, with the lowest correlation and highest error. Removing DEGs defined in other human datasets (-DEG Int.) yielded results that were close to the best case, indicating that simply filtering genes commonly differentially expressed between modalities across tissues recovers much of the benefit of dataset-specific DEG pruning.

When focusing in the Non-Removed Cells scenario, -DEG Int. yielded the highest Pearson correlation and an RMSE on par with

the snRNA-DEG reference (Figure 2E), suggesting that this gene list integrated across tissue types robustly reflects scRNA-seq and snRNA-seq differences. The random-gene control showed the highest RMSE again and surprisingly had comparable Pearson correlation mean to the -DEG of the other dataset's removal. Notably, the combined DEG list from other datasets was quite large, reducing the reference to only ∼6,000 genes in some cases. This may have discarded biologically informative markers needed for optimal deconvolution, and there are likely more sophisticated strategies to optimize filtering. The results are comparable to All Cells otherwise. It is worth noting that in the adipose dataset only, snRNA All (-DEG Int.) performed better than -Random Genes and -DEG Other Datasets (Figures S2D and S2E). We attribute this higher performance in the adipose dataset to this tissue type having fewer compartmental biases, i.e., less dramatic nuclear-to-cytoplasmic transcript variation[29,31] (Table S1). Of all tissues tested it shows, the lowest total number (and percentage) of DEGs, hinting at fewer nuclear-to-cytoplasmic transcript differences.

In the Removed Cells scenario, the random gene removal again performed as hypothesized, showing the lowest Pearson correlation and highest RMSE (Figure 2F). Interestingly, pruning the identified DEGs from other datasets produced the highest Pearson correlation of all strategies but was outperformed in RMSE by snRNA-DEG (Figure 2C). The DEGs of a specific dataset (snRNA-DEG) only include those of the cell types that are not removed, mimicking a real-life situation. The boost in performance in the Removed Cells scenario by -DEG Other Datasets suggests that removing genes with consistent differential expression across tissues may better generalize to recovering an absent cell type. This highlights that when a reference is incomplete, removing broadly conserved modality-biased genes may better generalize across tissues, whereas dataset-specific DEG removal more precisely calibrates the expression of available cell types. Overall, these findings reinforce that gene-set pruning, especially of dataset-specific or cross-dataset DEGs, can significantly enhance deconvolution when reference panels are incomplete, whereas indiscriminate gene removal offers little to no benefit. The performance metrics per dataset are shown in Figures S2D–S2F.

Due to the high performance of the -DEG Int. (i.e., genes that were found to be differentially expressed in at least one cell type in all three human datasets), we also analyzed the biological component involved with this list of genes. We hypothesized that the improvement in deconvolution performance by removing these genes is due to the high concordance of this list with intrinsic variations in cytoplasmic and nuclear RNA (i.e., scRNA-seq and snRNA-seq). We performed Gene Ontology (GO) analysis (see STAR Methods for details) and found that the statistically significant components were related to cytoplasmic or ribosomal components of the cell (Figure S3B). This supports the idea that the -DEG Int. list reflects true protocol-driven transcriptomic discrepancies rather than tissue-specific biology, explaining why it improves deconvolution across datasets. In Table S1, we include a table summary of the number of DEGs per cell type across all datasets, the percentage of total genes these DEGs constitute, and the number of genes in common between cell types for each of the datasets used, including those cell types in common.

Methodological robustness differentiates transformation strategies

We next aimed to test the transformed reference expression while deconvolving real bulks. Real bulk data do not have a reliable ground truth for proportions, so each reference yields arbitrary predicted proportions. Therefore, we used consistency in predictions as a measure of robustness, similar to previous work.[28] The idea is that if a transformed reference yields similar predicted proportions as the other transformations, it is likely to be reliable and robust transformation.[28] We employed a real-setting experimental design where we used all cells observed in 7 samples of scRNA-seq adipose tissue, and two cell types that are not observed in any scRNA-seq sample were added from 12 snRNA-seq samples. We transformed these 2 "missing" cell types, fat cells and neutrophils, with each of the transformations and added the transformed snRNA-seq cell to the scRNA-seq observed cell types to create one reference per transform. Therefore, the reference was composed of all scRNA-seq cells and transformed snRNA-seq. This is similar to what was done in the aforementioned pseudobulks vs. real bulks experiment (see STAR Methods for details).

We used each of these references (i.e., with each of the transformations) to deconvolve 434 real bulk samples. We compared the predicted proportions of each used reference with the predicted proportions of the other references (one vector of all predicted proportions vs. one vector of all predicted proportions) and computed the cosine similarity per transform pair.

The transform-to-transform similarities are summarized in a heatmap in Figure 3A. We computed the 95% CI of the mean cosine similarity per transform and show these distributions in Figure 3B. Overall, most transformations had high concordance in predicted proportions with each other, except for the PCA LS-DEG reference. This reduced similarity likely reflects PCA's sensitivity to the gene subset used in fitting. Because in PCA LS-DEG the model is fit on a DEG-filtered gene set, the scRNA vs. snRNA covariance structure, and thus the shift vector learned in PCA space, differs substantially from the structure used by other transforms. Surprisingly, removing the "intersection genes" (-DEG Int.), i.e., the set of genes found to be differentially expressed in all three human datasets, had the largest mean cosine similarity to the other transforms, reiterating the potential power of integrating the -DEGs across tissue types.

We then explore another form of robustness by using cells from a different patient in each reference and comparing similarity in predictions. Similarly, we expect that high concordance in the predicted proportions between different patients' cell types would signify high robustness in the transformation. We used snRNA-seq-derived adipocytes from 12 patients individually (same data as described earlier, just not combined) and transformed these cells with each of the described transformations. We used this reference per patient, per transform, to deconvolve the same 434 bulk samples (same data as described earlier). We calculated the cosine similarity, as described earlier, for each transformation across the predicted proportions of each patient's reference.

Most transforms achieved comparable cosine similarity (scVI-cond, -DEG Int., scVI LS, and -DEG Other Datasets), with snRNA all (-DEG Int.) and PCA LS-DEG achieving similar worse similarity

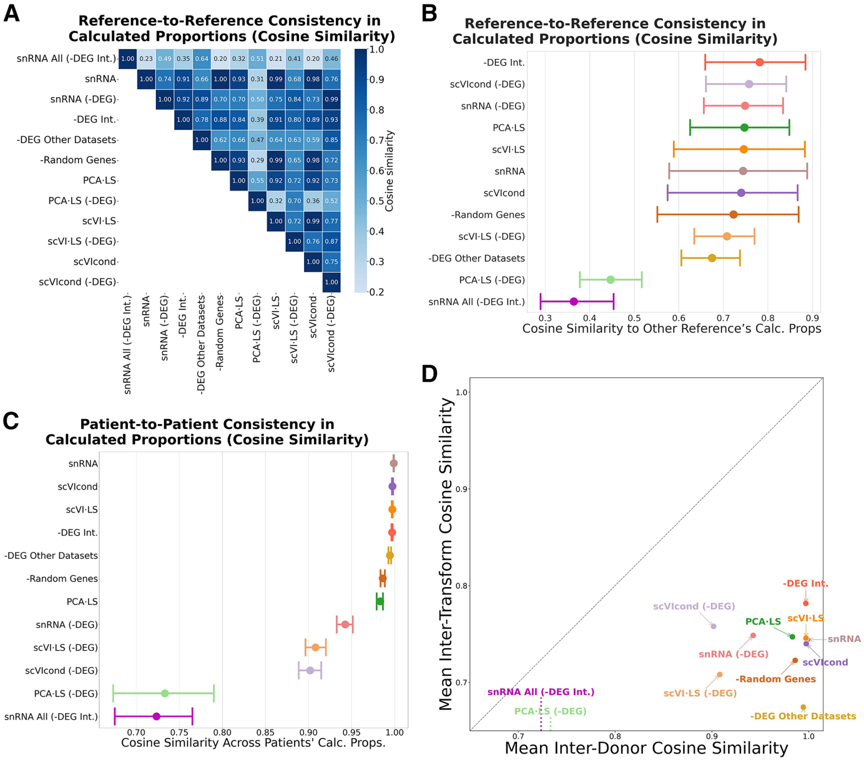

Figure 3. Evaluation of real adipose bulks deconvolved with each transformation and final scores per transform

(A) Heatmap showing the cosine similarity of calculated proportions per transformation—similarity between calculated proportions indicates reliability.

(B) The mean (dot) cosine similarity per transform, shown in (A), with 95% bootstrapped confidence intervals. Transforms ordered by mean value.

(C) Cosine similarities between calculated proportions using fat cells from different patients per transform, the mean value as a dot and the 95% bootstrapped confidence intervals shown. Transforms ordered by mean value.

(D) Composite robustness and accuracy scores including inter-transforms and inter-patient similarities and RMSE and Pearson correlation values, respectively. The transforms in the upper right quadrant have high robustness indicating reliability, and the axes are considered to yield the most robust and accurate results. *x* and *y* axes are truncated to highlight the variation.

scRNA-seq, single-cell RNA-sequencing; RMSE, root mean squared error; snRNA-seq, single-nucleus RNA-sequencing; DEG, differentially expressed gene.

(Figure 3C). Interestingly, adding raw snRNA-seq adipocytes to the reference yielded the highest mean (although comparable to the other transforms) of cosine similarity across all transformations. Also, all the transformations that include the removal of DEG had the lowest patient-to-patient concordance (Figure 3C). This pattern likely reflects that raw snRNA-seq adipocytes have highly stable nuclear expression profiles across donors,[29] leading to high concordance. In contrast, DEG-based methods rely on donor-specific DEG lists, and because each donor's snRNA-seq data contain slightly different cell compositions and detection biases, this causes each transform to operate on a different gene set and amplifies donor-specific variability, lowering patient-to-patient similarity. We observed in the snRNA-seq data that not all cell types appear in every donor, so each donor's DEG list is based on a different subset of cell populations. As a result, the genes we remove can differ wildly in identity and number across donors, making each donor-specific reference diverge rather than align.

We then combined both robustness measures: inter-transform (proportions predicted between transforms using same patient data) and inter-patient (proportions predicted between patients using same transform). We plotted each in an *x* and *y* axis, respectively, expecting the most robust transformation to have high values across both axes. We found one cluster of the most robust transforms that includes, in order, -DEG Int., scVI LS, scVIcond, snRNA-seq, and PCA LS. Surprisingly, the removal of random genes (-Random Genes) had high values across both axes too. We discuss this in the following section.

Joint accuracy-robustness scoring highlights well-performing transformation strategies across deconvolution methods

We next aimed to evaluate whether the deconvolution performance observed through BayesPrism across transformation strategies also generalized to other methods. We tested Scaden and SCDC, two methods with distinct methodologies (deep learning and weighted sums, respectively) that were previously found to perform well in deconvolution tasks.[32] We repeated all deconvolution experiments (pseudobulks and real bulks) using the two new methods. The detailed simulation experiment results can be found in the GitHub repository and Zenodo.[33,34] Each method was evaluated independently using the same set of transformations, yielding method-specific accuracy and robustness measurements.

To allow direct comparison across tools, we summarized each transformation into two composite scores per method: a simulation accuracy score integrating RMSE and Pearson correlation across all pseudobulks, and a robustness score integrating inter-transform and inter-patient consistency across real bulks (see STAR Methods). These summaries produced a two-dimensional accuracy-robustness landscape for each deconvolution method (Figure 4).

We observed that removal of the intersection genes (-DEG Int.) has the highest robustness score and the third highest accuracy score overall, further suggesting that this list of genes reflects true snRNA-seq to scRNA-seq differences that can aid deconvolution if removed from the reference. When evaluated across BayesPrism, Scaden, and SCDC, -DEG Int. consistently

CellPress
OPEN ACCESS

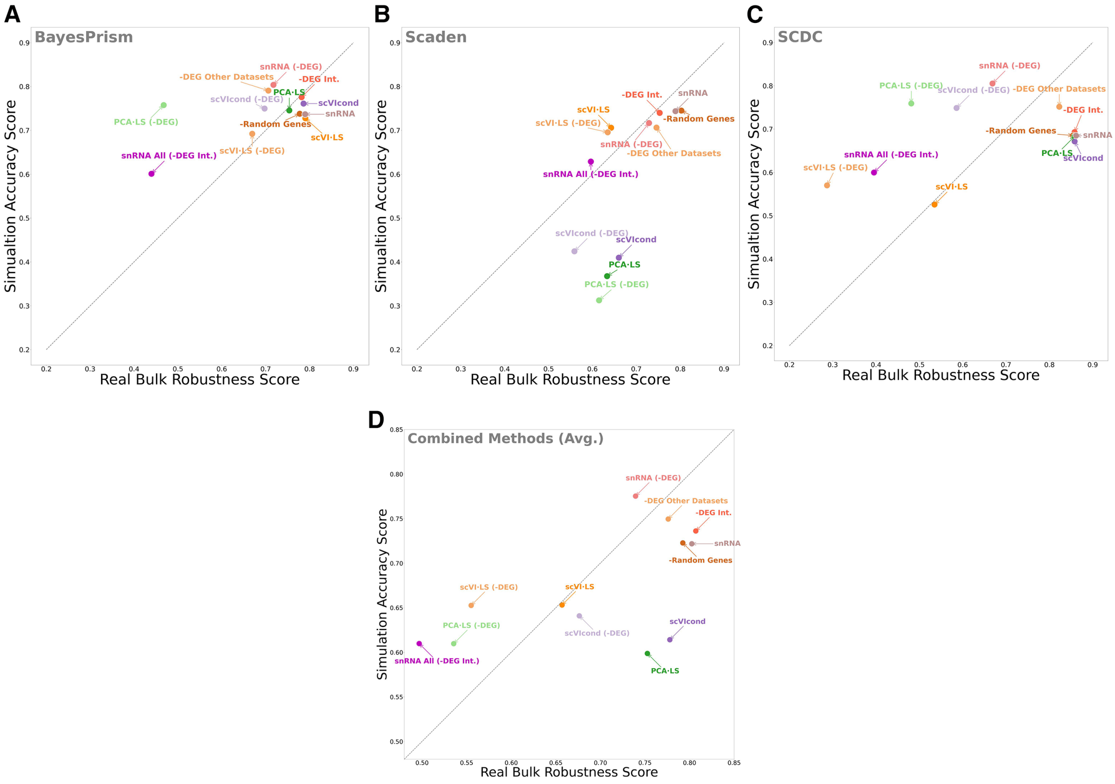

Figure 4. Accuracy-robustness scoring across three deconvolution methods

(A–C) Two-dimensional accuracy-robustness landscapes for all reference transformations evaluated using (A) BayesPrism, (B) Scaden, and (C) SCDC. For each method, simulation accuracy reflects the normalized mean of RMSE and Pearson correlation across all pseudobulk experiments, and robustness reflects the normalized mean cosine similarity across inter-transform and inter-patient comparisons from real bulk samples. Each point corresponds to one transformation applied to the scRNA-seq + snRNA-seq reference.

(D) Mean accuracy and robustness scores for each transformation averaged across BayesPrism, Scaden, and SCDC, providing an aggregated view of cross-method performance.

remained among the strongest-performing transformations, showing similarly high robustness and accuracy in all three methods as well as in the combined average. The scVIcond also has a high score across both axes, making it a very competitive alternative. This pattern was reproduced across all three deconvolution tools, where scVIcond again occupied the upper-right region of the accuracy-robustness space. The removal of the DEGs from the dataset (snRNA-DEG) had the highest accuracy, but because of the high donor-to-donor variability, it has the second worst robustness score, second to only the -DEG Other Datasets reference. We hypothesize again that this is due to the high data-to-data variability, and quantifying a different set of DEGs per dataset could give vastly different results depending on the number of matched cells. Across the three methods, snRNA-DEG showed this same pattern, very high accuracy but reduced robustness, supporting the idea that dataset-specific DEG lists vary substantially across donors and platforms.

The snRNA All (-DEG Int.) reference had the worst score in both accuracy and robustness. This highlights that even if the pruning of the -DEG Int. is shown to improve performance in mixed references (snRNA-seq added to scRNA-seq), it is not a viable alternative when using a full snRNA-seq reference. This result was consistent across BayesPrism, Scaden, and SCDC, where snRNA All (-DEG Int.) repeatedly fell at the bottom of both metrics.

Surprisingly, the removal of a random set of genes performed better than some of the more involved transformations (Figure 4). This effect is likely driven by the high redundancy and collinearity of single-cell transcriptomes: random dropout rarely removes key marker genes but preferentially eliminates noisy, low-information, or redundant features. As a result, it acts as an implicit regularization step, improving stability across donors and transformations despite its simplicity.[35] For the neural network-based Scaden model, this feature removal yields some of the highest accuracy and robustness (Figure 4B), again suggesting that this regularization is taking place through feature removal. This same trend appeared in all three methods and in the combined panel, where Random Gene removal again outperformed several structured transformations.

The strong performance of the -DEG Other Datasets transformation can be explained similarly. DEGs that replicate across external tissues are more likely to reflect consistent, protocol-driven differences between scRNA-seq and snRNA-seq, rather than donor-specific variability. Removing these cross-dataset DEGs therefore eliminates stable modality biases while avoiding

overfitting to idiosyncratic DEG calls, producing a cleaner and more generalizable correction across methods.

Overall, integrating results across BayesPrism, Scaden, and SCDC suggests that -DEG Int., -DEG Other Datasets, and snRNA-DEG consistently occupied the strongest accuracy-robustness region, with scVIcond as a robust alternative and Random Gene removal providing a surprisingly stable baseline.

DISCUSSION

The typical workflow for a researcher aiming to infer cell type proportions of bulk samples is to get an scRNA-seq sample (either new or available online) to use as a reference. If all cell types expected in bulk samples are observed in scRNA-seq, this workflow is expected to yield reliable proportions, yet the field now recognizes that this assumption often fails.[13,24]

Maden et al. showed that scRNA-seq and snRNA-seq capture different slices of the transcriptome and that mismatch alone can skew cell-fraction estimates.[36] They recommended performing controlled tests where the same tissue samples are profiled with both scRNA-seq and snRNA-seq and then deconvolved side-by-side, so that any errors caused by the protocol difference become obvious.[36] In the case where some cell types are missing, which we hypothesize is often, researchers have avoided this inconsistency by either using only snRNA-seq or scRNA-seq combined with snRNA-seq, to fill in the gap.[12,16,19,24] Another workaround has been building tissue-specific deconvolution models, such as *scNucConv*[21] and *DeTREM*,[22] for brain and human subcutaneous adipose tissue, respectively. However, these do not generalize beyond the tissues they were built for: even visceral adipose tissue had decreased performance for *snRNA-Conv* built for subcutaneous adipose tissue.[21]

SQUID[24] and *BISQUE*[23] can use either snRNA-seq or scRNA-seq as a reference and attacked the same problem from the bulk side: they leave the single-cell reference as is and instead mathematically reshape each bulk RNA-seq profile based on the reference. Our study breaks key assumptions the methods rely on, namely that the bulk and single-cell profiles be derived from the same patients or have the same cell type distribution, so we were unable to fairly evaluate them. Our study examines the integration of both modalities from different participants in a single reference and tackles the complementary reference side of the bias coin: we show that snRNA-only references are inadequate across four diverse tissues but that blending snRNA-seq with scRNA-seq restores and sometimes surpasses scRNA-only performance once you prune protocol-specific DEGs or apply deep learning-based transforms (e.g., scVIcond). Importantly, these trends were consistent across BayesPrism, Scaden, and SCDC, three methods with distinct modeling approaches, indicating that the transformation effects we observe are method agnostic rather than tool specific. Together, these works outline a full toolkit: adjust the bulk when the reference is trustworthy (*SQUID*/*BISQUE*), adjust or augment the reference when it is incomplete or protocol-mixed (our approaches, which can be used with any deconvolution method), and fall back on tissue-specific nuclei models only when neither option is feasible.

Our findings offer a clear cautionary tale: snRNA-seq references are not interchangeable with scRNA-seq references in deconvolution workflows. The substantial decrease in performance observed by using snRNA-seq as a reference vs. scRNA-seq, even with the same cell types in the same number, is a clear warning to researchers to refrain from using snRNA-seq as deconvolution references. We advise researchers to exclusively use an scRNA-seq reference for the cells that are available in scRNA-seq datasets and, when needed, append snRNA-seq cell types to the reference only using one of the transformations described in this study and not "as is." Even in the cases where the snRNA-seq cells are not of interest to enhance deconvolution accuracy overall, we recommend including snRNA-seq-exclusive cell types in the reference. In practice, this means that an scRNA-first strategy, supplemented with transformed snRNA-seq profiles when necessary, provides the most stable and generalizable performance.

We did not find a transformation that outperformed all others in all datasets, across all accuracy and robustness metrics, and the recommended transformation will depend on cell types present (scRNA-seq vs. snRNA-seq) and computing power available. We believe the heterogeneity between tissue types, cell types observed, tissue processing, and cell number will make a "one-size-fits-all" transformation a complicated endeavor. Across methods, we also observed a clear accuracy-robustness tradeoff: transformations such as snRNA-DEG delivered extremely high accuracy but reduced donor-to-donor stability, whereas -DEG Int. provided more balanced and repeatable performance. One key consideration in the choice of transformation is the data available: some transformations do not rely on matching cell types across scRNA-seq and snRNA-seq datasets (PCA LS, scVIcond, and scVI LS), making them a possible solution in cases where scRNA-seq and snRNA-seq observed cell types do not match. When the tissues being sequenced are small and there is only one sample for each modality, it could be difficult to find more than one matching cell type. The transformations that depend on matched cell types, namely the calculation and removal of dataset-specific DEGs, could be a viable solution when researchers have multiple batches and thus increased observed cell number. We also separated the deconvolution accuracy metrics by scenario (i.e., All Cells, Non-Removed Cells, and Removed Cells) (Figure 2) and by dataset (Figure S2) to encourage researchers to find the transformation with the best performance in the most relevant tissue type and scenario.

We found that including raw snRNA-seq adipocytes in the reference achieved the highest average patient-to-patient cosine similarity across transformations, though the improvement was small compared with other transforms. We hypothesize that this is because nuclear RNA tends to be enriched for unspliced, nascent transcripts or "housekeeping" gene programs,[37] which typically have low cell-to-cell or donor-to-donor variability, making a donor-to-donor comparison unexpectedly favor an unchanged snRNA-seq transcript. In contrast, our neighbor-based and VAE-based transforms likely accentuate biological and technical differences between batches and donors; thus, despite potentially improving deconvolution accuracy, this lowers the apparent patient-to-patient consistency.

A computationally reasonable alternative is removing the DEGs between scRNA-seq and snRNA-seq cell types, which made the biggest distinction in deconvolution performance across transformations in the simulations. In fact, adding snRNA-seq cell to an scRNA-seq reference and just removing DEGs with no other transformation outperformed some more computationally expensive transformations in some cases. However, this is not a one-size-fits-all transformation; we observe low donor-to-donor consistency in all transformations involving the removal of DEGs in real bulks. The identified DEGs greatly depend on which cell types are observed in the scRNA-seq and snRNA-seq datasets, since we calculate DEGs per matched cell type. This makes the removal of DEGs a less dependable strategy and should only be used in the cases where there are at least 4 cell types in common between snRNA-seq and scRNA-seq, and these cells are observed in reasonable numbers of more than 50. These are the parameters used in our simulations, showing high efficacy. Further work is required to evaluate whether fewer cell types or cell examples are a viable alternative.

If enough computing power is available, we recommend using scVIcond (i.e., a conditional implementation of the scVI VAE) as the first strategy. This transformation had high accuracy in simulations and high robustness (Figure 3E). Additionally, it does not depend on scRNA-seq and snRNA-seq having cell types in common for training, making it a feasible option in small datasets.

Our results indicate that removing the intersection of DEGs between 3 tissue types offers the most cost-effective path to the greatest deconvolution accuracy gain, making it the preferred strategy for integrating scRNA-seq with snRNA-seq samples. We found that -DEG Int. scored the highest consistency score and the second highest accuracy (second to only snRNA-DEG, which is third last in consistency). These high scores, along with the GO components observed, suggest that integrating multiple tissue types in the DEG analysis can further enhance the effect of scRNA-seq vs. snRNA-seq DEG removal. We have included this list of genes in the GitHub repository for download and use.[33] We encourage researchers to use and add onto this list with their own tissues of interest, which we hypothesize will further enhance the efficacy of this list. Nevertheless, this list is only applicable to human datasets, and further work is needed to find and validate an equivalent in other species.

Limitations of the study

Several limitations point the way forward for future studies. First, a key challenge in the space is that dissociating cells to create suspensions is, itself, a process that impacts cell abundance and expression. For example, even fluorescence-activated cell sorting (FACS) can induce composition biases similar to those in scRNA-seq.[38–40] In the long run, we anticipate that multiple modalities may need to be combined to identify the precise mixture of cells in a sample and their underlying expression patterns. Evaluating pseudobulks generated from alternative technologies, such as BD Rhapsody or SMART-seq, which capture a broader range of cell types, may help clarify mechanisms of cell type loss and further improve deconvolution strategies. Our evaluation is based on present-day solutions for widely used profiling methods and employing simulation to better understand performance in the context of a ground truth. Future work that integrates many complementary modalities, such as FACS, snRNA-seq, scRNA-seq, and spatial RNA expression profiling, may one day provide a more robust ground truth for evaluating deconvolution methods.

Second, the removal of DEGs between scRNA-seq and snRNA-seq cell types, although powerful and simple, could be enhanced further by including more datasets and cell types and optimizing thresholds. We also did not protect cell type marker genes, which might provide finer control. Second, we used scVI transformation types (scVI LS and scVIcond) with hyperparameters previously observed to perform well on alignment tasks,[25] but further tuning per tissue type could further enhance their accuracy. Third, the space of possible transformations (e.g., non-linear domain-adaptation nets, contrastive embeddings, and cross-modal diffusion models) is still largely unexplored, and methods purpose-built and hyper-parameter-tuned for this task may yet outperform the transformations we explored.

These results underscore the non-interchangeability of these modalities in deconvolution but also pave the way for future studies to compare the two in other scRNA-seq-targeted methods other than deconvolution (e.g., cell type annotation, batch normalization, deep learning models, etc.) rather than assuming snRNA-seq will perform equivalently.

Additionally, we tested only four tissues, and although the tested tissue types encompass a wide range, additional modality mismatches could be observed in other tissues that are hard to anticipate. Finally, our study focuses only on one deconvolution method, and while we do not expect results to vary greatly with other standard methods, future work should explore data integration in different deconvolution methodologies. We have included instructions on how to include a new dataset or transformation method in the GitHub repository[33] to allow researchers to quickly perform their own analyses. Together, we aim for our open-source framework and modality-aware guidelines to give the community a practical roadmap for turning vast bulk RNA-seq archives into cell-resolved insights.

RESOURCE AVAILABILITY

Lead contact

Further information and requests for resources and data should be directed to and will be fulfilled by the lead contact, Casey S. Greene (casey.s.greene@cuanschutz.edu).

Materials availability

This study did not generate new unique reagents.

Data and code availability

- The data that support the findings of this study are all freely available online. The count data for ADP and metastatic breast cancer (MBC) can be downloaded through the Gene Expression Omnibus with accession numbers GEO: GSE176067 (adipose single-cell),[41] GEO: GSE176171 (adipose single-nucleus),[31] GEO: GSE174475 (adipose bulks)[42] and GEO: GSE140819 (metastatic breast cancer).[43] The PBMC and MSB data provided by 10× Genomics can be directly accessed through their

website.[44] All data details and download links can be easily accessed in the GitHub repository.[33]

- The code developed for this study is available at our GitHub repository (https://github.com/greenelab/deconvolution_sc_sn_comparison),[33] under BSD 3-Clause License, and a Zenodo repository (https://doi.org/10.5281/zenodo.18165962).[34] The repository includes all necessary scripts and a README file with setup instructions and usage guidelines. For updates or assistance, users can refer to the repository or open an issue for queries. Our aim is to support transparency and reproducibility in computational research through this open-access resource.
- Any additional information required to reanalyze the data reported in this paper is available from the lead contact upon request.

STAR★METHODS

Detailed methods are provided in the online version of this paper and include the following:

- KEY RESOURCES TABLE
- EXPERIMENTAL MODEL AND STUDY PARTICIPANT DETAILS
- METHOD DETAILS
  - Datasets
  - Cell-type assignment
  - Data preprocessing and filtering for pseudobulks and references
  - Deconvolution of pseudobulks using references with transformed held-out cell types
  - Pseudobulks
  - Held-out cell reference and transformations
  - Transformations
  - Comparison of snRNA-seq transformed cell types with real scRNA-seq cells
  - Comparison of transformed cell types with real bulks
  - Deconvolution results and comparison
  - Deconvolution with DWLS and SCDC
  - Gene ontology analysis of intersection genes
  - Real adipose bulks data and single modality data preprocessing
  - Real bulks deconvolution robustness (per transform and per patient)
  - Composite score for each transform
- QUANTIFICATION AND STATISTICAL ANALYSIS

ACKNOWLEDGMENTS

This work was supported in part by a grant from the National Institutes of Health's National Cancer Institute (R01 CA237170).

AUTHOR CONTRIBUTIONS

Conceptualization, A.I. and C.S.G.; methodology, A.I. and C.S.G.; software, A.I. and C.S.G.; formal analysis, A.I. and C.S.G.; data curation, A.I. and C.S.G.; visualization, A.I. and C.S.G.; resources, A.I. and C.S.G.; writing – original draft, A.I. and C.S.G.; writing – review and editing, A.I. and C.S.G.; funding acquisition, C.S.G.

DECLARATION OF INTERESTS

The authors declare no competing interests.

DECLARATION OF GENERATIVE AI AND AI-ASSISTED TECHNOLOGIES IN THE WRITING PROCESS

During the preparation of this work, the authors used ChatGPT-4 to improve narrative flow and grammar. After using this tool, the authors reviewed and edited the content as needed and take full responsibility for the content of the publication.

SUPPLEMENTAL INFORMATION

Supplemental information can be found online at https://doi.org/10.1016/j.crmeth.2026.101346.

Received: September 15, 2025
Revised: December 3, 2025
Accepted: February 10, 2026
Published: March 26, 2026

REFERENCES

1. Wang, Z., Gerstein, M., and Snyder, M. (2009). RNA-Seq: a revolutionary tool for transcriptomics. Nat. Rev. Genet. *10*, 57–63. https://doi.org/10.1038/nrg2484.
2. Deshpande, D., Chhugani, K., Chang, Y., Karlsberg, A., Loeffler, C., Zhang, J., Muszyńska, A., Munteanu, V., Yang, H., Rotman, J., et al. (2023). RNA-seq data science: From raw data to effective interpretation. Front. Genet. *14*, 997383. https://doi.org/10.3389/fgene.2023.997383.
3. Hwang, H., Jeon, H., Yeo, N., and Baek, D. (2024). Big data and deep learning for RNA biology. Exp. Mol. Med. *56*, 1293–1321. https://doi.org/10.1038/s12276-024-01243-w.
4. Qiu, W., Dincer, A.B., Janizek, J.D., Celik, S., Pittet, M.J., Naxerova, K., and Lee, S.I. (2025). Deep profiling of gene expression across 18 human cancers. Nat. Biomed. Eng. *9*, 333–355. https://doi.org/10.1101/2024.03.17.585426.
5. Pividori, M., Lu, S., Li, B., Su, C., Johnson, M.E., Wei, W.-Q., Feng, Q., Namjou, B., Kiryluk, K., Kullo, I.J., et al. (2023). Projecting genetic associations through gene expression patterns highlights disease etiology and drug mechanisms. Nat. Commun. *14*, 5562. https://doi.org/10.1038/s41467-023-41057-4.
6. Li, X., and Wang, C.-Y. (2021). From bulk, single-cell to spatial RNA sequencing. Int. J. Oral Sci. *13*, 36. https://doi.org/10.1038/s41368-021-00146-0.
7. Tran, K.A., Addala, V., Johnston, R.L., Lovell, D., Bradley, A., Koufariotis, L.T., Wood, S., Wu, S.Z., Roden, D., Al-Eryani, G., et al. (2023). Performance of tumour microenvironment deconvolution methods in breast cancer using single-cell simulated bulk mixtures. Nat. Commun. *14*, 5758. https://doi.org/10.1038/s41467-023-41385-5.
8. Liao, J., Qian, J., Fang, Y., Chen, Z., Zhuang, X., Zhang, N., Shao, X., Hu, Y., Yang, P., Cheng, J., et al. (2022). De novo analysis of bulk RNA-seq data at spatially resolved single-cell resolution. Nat. Commun. *13*, 6498. https://doi.org/10.1038/s41467-022-34271-z.
9. Wang, X., Park, J., Susztak, K., Zhang, N.R., and Li, M. (2019). Bulk tissue cell type deconvolution with multi-subject single-cell expression reference. Nat. Commun. *10*, 380. https://doi.org/10.1038/s41467-018-08023-x.
10. Chu, T., Wang, Z., Pe'Er, D., and Danko, C.G. (2022). Cell type and gene expression deconvolution with BayesPrism enables Bayesian integrative analysis across bulk and single-cell RNA sequencing in oncology. Nat. Cancer *3*, 505–517. https://doi.org/10.1038/s43018-022-00356-3.
11. Newman, A.M., Steen, C.B., Liu, C.L., Gentles, A.J., Chaudhuri, A.A., Scherer, F., Khodadoust, M.S., Esfahani, M.S., Luca, B.A., Steiner, D., et al. (2019). Determining cell type abundance and expression from bulk tissues with digital cytometry. Nat. Biotechnol. *37*, 773–782. https://doi.org/10.1038/s41587-019-0114-2.
12. Hippen, A.A., Davidson, N.R., Barnard, M.E., Weber, L.M., Gertz, J., Doherty, J.A., Hicks, S.C., and Greene, C.S. (2023). Deconvolution Reveals Compositional Differences in High-Grade Serous Ovarian Cancer Subtypes (Cold Spring Harbor Laboratory).
13. Ivich, A., Davidson, N.R., Grieshober, L., Li, W., Hicks, S.C., Doherty, J.A., and Greene, C.S. (2025). Missing cell types in single-cell references impact deconvolution of bulk data but are detectable. Genome Biol. *26*, 86. https://doi.org/10.1186/s13059-025-03506-9.

14. Twa, G.M., Phillips, R.A., Robinson, N.J., and Day, J.J. (2025). Accurate Sample Deconvolution of Pooled snRNA-Seq Using Sex-dependent Gene Expression Patterns. NAR Genomics and Bioinformatics *7*. https://doi.org/10.1093/nargab/lqaf156.

15. Sosina, O.A., Tran, M.N., Maynard, K.R., Tao, R., Taub, M.A., Martinowich, K., Semick, S.A., Quach, B.C., Weinberger, D.R., Hyde, T., et al. (2021). Strategies for cellular deconvolution in human brain RNA sequencing data. F1000Res. *10*, 750. https://doi.org/10.12688/f1000research.50858.1.

16. Conning-Rowland, M., Cheng, C.W., Brown, O., Giannoudi, M., Levelt, E., Roberts, L.D., Griffin, K.J., and Cubbon, R.M. (2025). Application of CIBERSORTx and BayesPrism to deconvolution of bulk RNA-seq data from human myocardium and skeletal muscle. Heliyon *11*, e42499. https://doi.org/10.1016/j.heliyon.2025.e42499.

17. Sutton, G.J., Poppe, D., Simmons, R.K., Walsh, K., Nawaz, U., Lister, R., Gagnon-Bartsch, J.A., and Voineagu, I. (2022). Comprehensive evaluation of deconvolution methods for human brain gene expression. Nat. Commun. *13*, 1358. https://doi.org/10.1038/s41467-022-28655-4.

18. Wu, H., Kirita, Y., Donnelly, E.L., and Humphreys, B.D. (2019). Advantages of Single-Nucleus over Single-Cell RNA Sequencing of Adult Kidney: Rare Cell Types and Novel Cell States Revealed in Fibrosis. J. Am. Soc. Nephrol. *30*, 23–32. https://doi.org/10.1681/asn.2018090912.

19. Huuki-Myers, L.A., Montgomery, K.D., Kwon, S.H., Cinquemani, S., Eagles, N.J., Gonzalez-Padilla, D., Maden, S.K., Kleinman, J.E., Hyde, T.M., Hicks, S.C., et al. (2025). Benchmark of Cellular Deconvolution Methods Using a Multi-Assay Reference Dataset from Postmortem Human Prefrontal Cortex. Genome Biol. *26*. https://doi.org/10.1186/s13059-025-03552-3.

20. Emont, M.P., Jacobs, C., Essene, A.L., Pant, D., Tenen, D., Colleluori, G., Di Vincenzo, A., Jørgensen, A.M., Dashti, H., Stefek, A., et al. (2022). A single-cell atlas of human and mouse white adipose tissue. Nature *603*, 926–933. https://doi.org/10.1038/s41586-022-04518-2.

21. Sorek, G., Haim, Y., Chalifa-Caspi, V., Lazarescu, O., Ziv-Agam, M., Hagemann, T., Nono Nankam, P.A., Blüher, M., Liberty, I.F., Dukhno, O., et al. (2024). sNucConv: A bulk RNA-seq deconvolution method trained on single-nucleus RNA-seq data to estimate cell-type composition of human adipose tissues. iScience *27*, 110368. https://doi.org/10.1016/j.isci.2024.110368.

22. O'Neill, N.K., Stein, T.D., Hu, J., Rehman, H., Campbell, J.D., Yajima, M., Zhang, X., and Farrer, L.A. (2023). Bulk brain tissue cell-type deconvolution with bias correction for single-nuclei RNA sequencing data using DeTREM. BMC Bioinf. *24*, 349. https://doi.org/10.1186/s12859-023-05476-w.

23. Jew, B., Alvarez, M., Rahmani, E., Miao, Z., Ko, A., Garske, K.M., Sul, J.H., Pietiläinen, K.H., Pajukanta, P., and Halperin, E. (2020). Accurate estimation of cell composition in bulk expression through robust integration of single-cell information. Nat. Commun. *11*, 1971. https://doi.org/10.1038/s41467-020-15816-6.

24. Cobos, F.A., Panah, M.J.N., Epps, J., Long, X., Man, T.-K., Chiu, H.-S., Chomsky, E., Kiner, E., Krueger, M.J., Di Bernardo, D., et al. (2023). Effective methods for bulk RNA-seq deconvolution using scnRNA-seq transcriptomes. Genome Biol. *24*, 177. https://doi.org/10.1186/s13059-023-03016-6.

25. The scvi-tools development team (2025). Atlas-level integration of lung data. https://docs.scvi-tools.org/en/stable/tutorials/notebooks/scrna/harmonization.html.

26. Gayoso, A., Lopez, R., Xing, G., Boyeau, P., Valiollah Pour Amiri, V., Hong, J., Wu, K., Jayasuriya, M., Mehlman, E., Langevin, M., et al. (2022). A Python library for probabilistic analysis of single-cell omics data. Nat. Biotechnol. *40*, 163–166. https://doi.org/10.1038/s41587-021-01206-w.

27. Moras, M., Lefevre, S.D., and Ostuni, M.A. (2017/12/19). Frontiers | From Erythroblasts to Mature Red Blood Cells: Organelle Clearance in Mammals. Front. Physiol. *8*, 1076. https://doi.org/10.3389/fphys.2017.01076.

28. Hippen, A.A., Omran, D.K., Weber, L.M., Jung, E., Drapkin, R., Doherty, J.A., Hicks, S.C., and Greene, C.S. (2023). Performance of computational algorithms to deconvolve heterogeneous bulk ovarian tumor tissue depends on experimental factors. Genome Biol. *24*, 239. https://doi.org/10.1186/s13059-023-03077-7.

29. Gupta, A., Shamsi, F., Altemose, N., Dorlhiac, G.F., Cypess, A.M., White, A.P., Yosef, N., Patti, M.E., Tseng, Y.-H., and Streets, A. (2022). Characterization of transcript enrichment and detection bias in single-nucleus RNA-seq for mapping of distinct human adipocyte lineages. Genome Res. *32*, 242–257. https://doi.org/10.1101/gr.275509.121.

30. Yao, Z., Liu, H., Xie, F., Fischer, S., Adkins, R.S., Aldridge, A.I., Ament, S.A., Bartlett, A., Behrens, M.M., Van Den Berge, K., et al. (2021). A transcriptomic and epigenomic cell atlas of the mouse primary motor cortex. Nature *598*, 103–110. https://doi.org/10.1038/s41586-021-03500-8.

31. Rosen, E.D., Tsai, L.T., and Emont, M.P. (2022). A single cell atlas of human adipose tissue. https://www.ncbi.nlm.nih.gov/geo/query/acc.cgi?acc=GSE176171.

32. Dietrich, A., Merotto, L., Pelz, K., Eder, B., Zackl, C., Reinisch, K., Edenhofer, F., Marini, F., Sturm, G., List, M., and Finotello, F. (2026). Omnideconv: A Unifying Framework for Using and Benchmarking Single-Cell-Informed Deconvolution of Bulk RNA-Seq Data. Genome Biol. *27*. https://doi.org/10.1186/s13059-026-03955-w.

33. Ivich, A. (2025). Comparison of single-nucleus and single-cell as deconvolution references and potential transformations. https://github.com/greenelab/deconvolution_sc_sn_comparison.

34. Ivich, A. (2026). greenelab/deconvolution_sc_sn_comparison: Publication for "Integrating single-cell and single-nucleus datasets improves bulk RNA-seq deconvolution" (v1). https://doi.org/10.5281/zenodo.18165963.

35. Venet, D., Dumont, J.E., and Detours, V. (2011). Most Random Gene Expression Signatures Are Significantly Associated with Breast Cancer Outcome. PLoS Comput. Biol. *7*, e1002240. https://doi.org/10.1371/journal.pcbi.1002240.

36. Maden, S.K., Kwon, S.H., Huuki-Myers, L.A., Collado-Torres, L., Hicks, S.C., and Maynard, K.R. (2023). Challenges and opportunities to computationally deconvolve heterogeneous tissue with varying cell sizes using single-cell RNA-sequencing datasets. Genome Biol. *24*, 288. https://doi.org/10.1186/s13059-023-03123-4.

37. Lake, B.B., Codeluppi, S., Yung, Y.C., Gao, D., Chun, J., Kharchenko, P.V., Linnarsson, S., and Zhang, K. (2017). A comparative strategy for single-nucleus and single-cell transcriptomes confirms accuracy in predicted cell-type expression from nuclear RNA. Sci. Rep. *7*, 6031. https://doi.org/10.1038/s41598-017-04426-w.

38. Ikeuchi, T., Akhi, R., Cardona Rodriguez, B., Fraser, D., Williams, D., Kim, T.S., Greenwell-Wild, T., Overmiller, A., Morasso, M., and Moutsopoulos, N. (2024). Dissociation of murine oral mucosal tissues for single cell applications. J. Immunol. Methods *525*, 113605. https://doi.org/10.1016/j.jim.2023.113605.

39. Mason, E. (2023). Immunophenotyping by Flow Cytometry. https://www.biocompare.com/Editorial-Articles/597499-Immunophenotyping-by-Flow-Cytometry/.

40. Lorenzo, J.V. (2025). Tissue Dissociation: The First Step Toward Single Cell Analysis. https://singleron.bio/what-is-tissue-dissociation/.

41. Rosen, E.D., and Tsai, L.T. (2022). Characterization of the stromal vascular fraction (SVF) of human subcutaneous adipose tissue (SAT). https://www.ncbi.nlm.nih.gov/geo/query/acc.cgi?acc=GSE176067.

42. Tsai, L., and Rosen, E. (2022). Epigenomic and Transcriptional Basis of Human InsulinResistance. https://www.ncbi.nlm.nih.gov/geo/query/acc.cgi?acc=GSE174475.

43. Slyper, M., Porter, C.B.M., Ashenberg, O., Waldman, J., Drokhlyansky, E., Wakiro, I., Smillie, C., Smith-Rosario, G., Wu, J., Dionne, D., et al. (2020). A single-cell and single-nucleus RNA-Seq toolbox for fresh and frozen human tumors. Nat. Med. *26*, 792–802. https://doi.org/10.1038/s41591-020-0844-1.

44. 10x Genomics. Datasets. https://www.10xgenomics.com/datasets?configure%5BhitsPerPage%5D=50&configure%5BmaxValuesPerFacet%5D=1000
45. Domínguez Conde, C., Xu, C., Jarvis, L.B., Rainbow, D.B., Wells, S.B., Gomes, T., Howlett, S.K., Suchanek, O., Polanski, K., King, H.W., et al. (2022). Cross-tissue immune cell analysis reveals tissue-specific features in humans. Science *376*. https://doi.org/10.1126/science.abl5197.
46. Eden, E., Navon, R., Steinfeld, I., Lipson, D., and Yakhini, Z. (2009). GOrilla: a tool for discovery and visualization of enriched GO terms in ranked gene lists. BMC Bioinformatics *10*, 48. https://doi.org/10.1186/1471-2105-10-48.
47. Hu, M., and Chikina, M. (2024). InstaPrism: An R Package for Fast Implementation of BayesPrism. Bioinformatics *40*. https://doi.org/10.1093/bioinformatics/btae440.
48. Menden, K., Marouf, M., Oller, S., Dalmia, A., Magruder, D.S., Kloiber, K., Heutink, P., and Bonn, S. (2020). Deep learning–based cell composition analysis from tissue expression profiles. Sci. Adv. *6*, eaba2619. https://doi.org/10.1126/sciadv.aba2619.
49. Dong, M., Thennavan, A., Urrutia, E., Li, Y., Perou, C.M., Zou, F., and Jiang, Y. (2021). SCDC: bulk gene expression deconvolution by multiple single-cell RNA sequencing references. Brief. Bioinform. *22*, 416–427. https://doi.org/10.1093/bib/bbz166.
50. La Manno, G., Siletti, K., Furlan, A., Gyllborg, D., Vinsland, E., Mossi Albiach, A., Mattsson Langseth, C., Khven, I., Lederer, A.R., Dratva, L.M., et al. (2021). Molecular architecture of the developing mouse brain. Nature *596*, 92–96. https://doi.org/10.1038/s41586-021-03775-x.
51. Wilk, A.J., Rustagi, A., Zhao, N.Q., Roque, J., Martínez-Colón, G.J., McKechnie, J.L., Ivison, G.T., Ranganath, T., Vergara, R., Hollis, T., et al. (2020). A single-cell atlas of the peripheral immune response in patients with severe COVID-19. Nat. Med. *26*, 1070–1076. https://doi.org/10.1038/s41591-020-0944-y.
52. Lun, A.T., L., Bach, K., and Marioni, J.C. (2016). Pooling across cells to normalize single-cell RNA sequencing data with many zero counts. Genome Biol. *17*. https://doi.org/10.1186/s13059-016-0947-7.
53. Muzellec, B., Teleńczuk, M., Cabeli, V., and Andreux, M. (2023). PyDESeq2: a python package for bulk RNA-seq differential expression analysis. Bioinformatics *39*. https://doi.org/10.1093/bioinformatics/btad547.
54. Haghverdi, L., Lun, A.T.L., Morgan, M.D., and Marioni, J.C. (2018). Batch effects in single-cell RNA-sequencing data are corrected by matching mutual nearest neighbors. Nat. Biotechnol. *36*, 421–427. https://doi.org/10.1038/nbt.4091.
55. Mudge, J.M., Carbonell-Sala, S., Diekhans, M., Martinez, J.G., Hunt, T., Jungreis, I., Loveland, J.E., Arnan, C., Barnes, I., Bennett, R., et al. (2025). GENCODE 2025: reference gene annotation for human and mouse. Nucleic Acids Res. *53*, D966–D975. https://doi.org/10.1093/nar/gkae1078.

CellPress
OPEN ACCESS

STAR★METHODS

KEY RESOURCES TABLE

REAGENT or RESOURCE	SOURCE	IDENTIFIER
Deposited data		
Mouse embryonic brain single-nucleus RNA-seq reference dataset	10x Genomics[44]	https://www.10xgenomics.com/datasets/5-k-mouse-e-18-combined-cortex-hippocampus-and-subventricular-zone-nuclei-3-1-standard-6-0-0
Mouse embryonic brain single-cell RNA-seq reference dataset	10x Genomics[44]	https://www.10xgenomics.com/datasets/9-k-brain-cells-from-an-e-18-mouse-2-standard-2-0-1
Human PBMC single-nucleus RNA-seq reference dataset	10x Genomics[44]	https://www.10xgenomics.com/datasets/10-k-human-pbm-cs-multiome-v-1-0-chromium-x-1-standard-2-0-0
Human PBMC single-cell RNA-seq reference dataset	10x Genomics[44]	https://www.10xgenomics.com/datasets/10-k-human-pbm-cs-5-v-2-0-chromium-x-2-standard-6-1-0
Human adipose tissue single-nucleus RNA-seq dataset	Original study[31]	GEO: GSE176171
Human adipose tissue single-cell RNA-seq dataset	Original study[41]	GEO: GSE176067
Human adipose tissue bulk RNA-seq dataset	Original study[42]	GEO: GSE174475
Metastatic breast cancer liver single-cell and single-nucleus RNA-seq dataset	Original study[43]	GEO: GSE140819
Software and algorithms		
Python	Python Software Foundation	v3.10.16
R	R Foundation for Statistical Computing	v4.3.3
scvi-tools	Gayoso et al.[26]	v1.2.2.post2
CellTypist	Domínguez Conde et al.[45]	v1.7.1
GOrilla (Gene Ontology enrichment analysis tool)	Eden et al.[46]	http://cbl-gorilla.cs.technion.ac.il/
omnideconv (R package; used for Scaden and SCDC runs)	Dietrich et al.[32]	https://github.com/omnideconv/omnideconv (v1)
BayesPrism	Chu et al.[10]	https://github.com/ding-lab/BayesPrism
InstaPrism (BayesPrism implementation)	Hu and Chikina[47]	https://github.com/humengying0907/InstaPrism
Scaden	Menden et al.[48]	https://github.com/KrishnaswamyLab/scaden
SCDC	Dong et al.[49]	https://github.com/meichendong/SCDC
Other		
Analysis code repository	This paper	https://github.com/greenelab/deconvolution_sc_sn_comparison
Archived code and processed results	This paper	https://zenodo.org/records/18165963

EXPERIMENTAL MODEL AND STUDY PARTICIPANT DETAILS

This study did not generate new experimental data or enroll new human or animal subjects. All analyses were performed using publicly available, de-identified human and mouse datasets, and ethical approval, informed consent, and animal care procedures were obtained by the original studies from which the data were derived.

METHOD DETAILS

Datasets

This study utilized exclusively publicly available datasets. The datasets used for the pseudobulk simulation experiment consist of one snRNA-seq and two scRNA-seq batches (see data and code availability section for access details). To ensure a diverse and representative sample, we used a variety of tissue types with variable dissociation-induced bias or cell loss from diverse sources. Specifically, we used data from two non-pathological human tissues: one with dissociation bias based on prior evidence (adipose tissue) and one with minimal hypothesized dissociation bias (PBMC tissue) from 10x Genomics. We also used a cancerous tissue, metastatic breast cancer, which have significant differences compared to non-pathological tissues. Lastly, we included a E16 (in development) mouse brain tissue to see the applicability in datasets from nonhuman species and in early-stage differentiation.

Each of the datasets consists of 3 batches: one scRNA-seq dataset to create pseudobulks, an independent (not same donor) scRNA-seq dataset for single-cell references, and one snRNA-seq reference to add to scRNA-seq deconvolution reference. We use the word "batch" or "sample" to mean one independent dataset from one donor only. In the case of the MBC, the 2 reference datasets come from the same patient. For the ADP, PBMC and MSB data, the two reference datasets come from different donor/mice and protocols. In all cases, the reference datasets and the pseudobulk reference are not the same patient.

For the real bulk analysis, we used 434 real bulk RNA-seq samples, and an additional 12 patient samples of snRNA-seq and 7 samples of scRNA-seq.

Cell-type assignment

For both the ADP and MBC datasets, cell types were assigned in the original publication, and those cell types were used for the analysis. For the PBMC and MSB datasets, we assigned cell types using CellTypist,[45] a logistic regression based assignment to ensure consistency and reproducibility. We used the "developing mouse brain" v1[50] model for MSB and the "Healthy COVID19 PBMC" v1 models[51] for PBMC.

Data preprocessing and filtering for pseudobulks and references

All datasets used are publicly available and previously processed. We added a file to the GitHub repository[33] that contains details and links for all data sources for each dataset used in this study, as well as any additional filtering parameters when needed.

For each data type (ADP, PBMC, MBC and MSB) we removed all cell types with less than 50 cells in either of the datasets to ensure sufficient variability for pseudobulks and references in the simulation studies. We also aligned the gene expression matrices of the scRNA-seq and snRNA-seq datasets to include only the common genes between them.

Deconvolution of pseudobulks using references with transformed held-out cell types

After data and cell type filtering, we created pseudobulks with known proportions and deconvolved them with various reference types. This process was the same for all datasets (ADP, PBMC, MBC and MSB). We created pseudobulks with all available cell types using one of the scRNA-seq datasets. In order to mimic what happens in a true research setting, we removed one cell type at a time ("held-out") from each of the scRNA-seq references and replaced this cell type with the same cell type but from snRNA-seq (nuclear RNA only) either "raw" (no change to the data) or with different transformations to harmonize the expression profiles of the held-out snRNA-seq cell type with the rest of the scRNA-seq reference (see below). We created two control references: one with all scRNA-seq cells (positive control), and one of all snRNA-seq cells (negative control), both matching all cell types present in the pseudobulks. For all references and in all transformations, the number of cell examples per cell type was kept the same.

For each held-out cell type, we trained a separate scVI model that excluded that cell type from both the scRNA-seq training set and the PCA fitting step. This approach mimics real-world scenarios in which certain cell types are missing from the reference dataset, ensuring that all transformations remain valid and that our results closely reflect practical performance.

Pseudobulks

We created pseudobulks for each of our datasets (ADP, PBMC, MSB, and MBC). All pseudobulks only contain scRNA-seq cells from the pseudobulk-dataset, not used in the scRNA-seq reference and independent of the scRNA-seq reference (i.e., not the same patient). We used a custom sampling strategy to create pseudobulks of known proportions. For each pseudobulk sample, a cell-type proportion vector was generated according to one of two schemes:

- **Random Proportions:** A Dirichlet distribution (with equal concentration parameters) was used to generate a random proportion vector. In cases where the resulting cell counts (i.e., the product of the proportion vector and a fixed number of cells 1,000) resulted in zero for any cell type, the sampling was repeated until every cell type was represented.
- **Realistic Proportions:** The empirical observed cell-type proportions (as computed from the scRNA-seq data) was used as the base proportion, with a small normally distributed noise (mean = 0, SD = 0.01) added. The resulting noisy vector was normalized to add up to 1, and cell counts were computed as described for the random case. Again, resampling was performed if any cell type was assigned zero cells.

CellPress
OPEN ACCESS

For each pseudobulk sample, the required number of cells from each cell type (as determined by the proportion vector) was randomly sampled from the scRNA-seq data without replacement when possible (i.e., when enough cells of that type are available). These gene expression profiles were then summed. To mimic technical variation, Gaussian noise (mean = 0; SD = 0.05) was added to each pseudobulk, and negative expression values were clipped to zero. A total of 500 pseudobulks were generated under each proportion type (realistic and random), yielding 1,000 pseudobulk samples overall. These pseudobulks, for which we had corresponding cell-type ground truth proportion matrices, were used as input to the deconvolution method.

Held-out cell reference and transformations

For each cell type in our datasets (ADP, PBMC, MBC and MSB), we replaced the scRNA-seq expression with either a "raw" snRNA-seq counterpart, or that same snRNA-seq expression with different transformations (outlined below). We deconvolved the same 1000 pseudobulks with each of the reference types. We also included the positive control (i.e., held-out cell is not replaced, just scRNA-seq).

Transformations

- **Non-Transformed: Controls (scRNA All (PosCtrl), snRNA All (NegCtrl), snRNA-seq):** In the pseudobulk simulation experiments, we created the following controls to compare the performance of the transformations below. For each dataset, we created one reference that includes all cell types available in the pseudobulk, where all cells come from a scRNA-seq dataset (labeled scRNA All (PosCtrl)). This reference represents the ideal research scenario and positive control, although not realistic in some circumstances. We also created one reference of equal cell types and number of cells per cell type, but where all cells come from a snRNA-seq dataset, labeled (snRNA All (NegCtrl). For the experiments in which we hold-out one scRNA-seq cell, we also created one reference per cell type where remove one cell type at a time from the scRNA All (PosCtrl) reference and replace that cell's expression with the equivalent from the snRNA All (NegCtrl) reference (labeled snRNA-seq).

In the real ADP data experiments with real bulks, using a reference will all cell types in scRNA-seq or all cell types in snRNA-seq without integration would not be comparable; the datasets do not have matched cell types (i.e., one and two cell types missing) even when we integrate multiple datasets from each. We created the "snRNA-seq" reference by combining all the cells available in scRNA-seq datasets and added the remaining cell types (missing from scRNA-seq) from snRNA-seq datasets, mimicking a real-world scenario.

- **Differentially expressed genes removed (snRNA All (-DEG Int.), snRNA -DEG, -Random Genes, -DEG Other Datasets, -DEG Int.):** To calculate the differentially expressed genes between scRNA-seq and snRNA-seq per cell type, we created S-cell aggregated and snRNA-seq aggregated (sum of the expression of 10 cells) for each cell type as recommended in.[52] We used this cell type aggregates to compute the DEG using pyDESEQ2 version 0.5.0.[53] DEGs were defined as those with a p-adjusted value of less than 0.01 after Benjamini-Hochberg adjustment. We removed the union of DEGs of all cell types (per dataset) from some of the cell references (labeled snRNA -DEG), therefore removing them from the deconvolution process.

We tested the removal of other gene groups in deconvolution. These reference test whether pruning improves alignment of the bulk and reference, and therefore improve deconvolution. For the snRNA All (-DEG Int.) reference, created only for human datasets, we used all snRNA-seq cells (no held out), and removed the list of genes that was found to be differentially expressed in all human datasets (the intersection or Int.). We additionally created references with a random set of genes removed of the same number as the DEGs (labeled -Random) to validate the biological context of the calculated -DEGs. For the -Random Genes control, we sampled a size-matched set of genes uniformly from the genes present in the reference and bulk feature space after excluding (i) the union of per-dataset scRNA-seq vs snRNA-seq DEGs and (ii) the cross-dataset intersection genes (-DEG Int.). For the human datasets, we also created references with the intersection DEGs removed (-DEG Int.) (similar to snRNA All (-DEG Int.), but all scRNA-se cells with added snRNA-seq), and removing the DEGs that were found in the other datasets not including the current data (e.g., removing the DEGs from ADP and PBMC in MBC) (labeled -DEG Other Datasets). The details on the references, genes removed, and cells included are outlined in Table 1.

- **PCA neighbour-based shift (PCA·LS and PCA·LS -DEG):** For each dataset (ADP, PBMC, MSB, and MBC), cell counts for scRNA-seq and overlapping cell types in snRNA-seq were first transformed with log $(x + 1)$ and then standardized to zero mean and unit variance. Principal-component analysis was fitted to the scaled data, retaining the minimum number of components required to capture at least 75% of the total variance. The scRNA-seq and overlapping snRNA-seq cells were projected into this lower dimensional PCA space. We shift the cell's expression similar to what is done in[54]: we first found the centroid of all scRNA-seq observations. For each (only overlapping) snRNA-seq cell, we calculated an observation-specific shift as the distance between that cell and the scRNA-seq centroid, giving us a list of distances for each overlapping snRNA-seq cell. Then, the held-out or "missing" cell type (for which we do not have a scRNA-seq example) cells were projected into the same fitted PCA space. For each cell example of this "missing" cell type, we calculated the Euclidian distance to other snRNA-seq cells and found the 10 nearest neighbours in the PCA space. We then used the mean distance of these 10 nearest neighbours as a shift vector for each cell, and this shift was added to each cell according to its nearest neighbours, shifting the snRNA-seq

expression. Finally, the shifted latent representations were back-projected to gene expression space by applying the inverse PCA·LS transform, followed by taking the natural exponential and then subtracting 1 to revert the log-transformation, and subsequently scaled to match the median library size of the scRNA-seq data (mean count value). Each of these steps was repeated for the datasets with the DEGs removed (labeled PCA·LS -DEG).

- **scVI (VAE) model with latent space shift (scVI·LS and scVI·LS -DEG):** For each dataset (ADP, PBMC, MSB, and MBC), we used a traditional scVI VAE model to "transform" one data type (snRNA-seq) to another (scRNA-seq) through alignment in the latent space. We trained multiple scVI models for each data type: one for each removed cell, ensuring the training data never contained the held-out or "missing" cell type to simulate a real life scenario.[13] For all models, we used 2 layers, 30 latent variables, gene-batch dispersion and negative binomial gene likelihood (default parameters otherwise) because these parameters have shown to work well in alignment tasks,[25] with no conditional encoding of data type. All models were trained with an early stopping patience of 10 epochs. All code showing model training can be found in the GitHub repository under scripts/train_scvi_models_allgenes.py and scripts/train_scvi_models_nodeg.py without DEGs.[33] After training, we encoded the snRNA-seq held-out or "missing" cells to the latent space and shifted each cell the same way it was done in the PCA neighbour-based shift described above. We then decoded the shifted expression and used the median library size from the scRNA-seq cells to scale the decoded snRNA-seq data, as is described above for the PCA shift. We repeated this reference with and without the DEGs (i.e., new models trained with less gene features) as computed above.
- **scVI (VAE) conditional model (scVIcond and scVIcond -DEG):** For each dataset (ADP, PBMC, MSB, and MBC), we used a conditional scVI VAE model to "transform" one data type (snRNA-seq) to another (scRNA-seq). We trained multiple scVI models for each data type: one for each removed cell, ensuring the training data never contained the held-out or "missing" cell type to simulate a real-life scenario.[13] All models were set to be conditional on the data type, either scRNA-seq or snRNA-seq, by one-hot encoding the data type label as a feature (encoded at encoder and injected at latent space). For all datasets and cell types, we used 2 layers, 30 latent variables, gene-batch dispersion and negative binomial gene likelihood (default parameters otherwise) because these parameters have shown to work well in alignment tasks.[25] The training parameters were kept constant and the same as described above for scVI (VAE) latent space neighbour-based shift. All code showing model training can be found in the GitHub repository under scripts/train_scvi_models_allgenes.py and scripts/train_scvi_models_nodeg.py without DEGs.[33] After training, we encoded and decoded the snRNA-seq removed cell with scRNA-seq labels, which we hypothesize would cause the model to transform the snRNA-seq expression into a scRNA-seq counterpart. We used the median library size from the scRNA-seq cells to scale the decoded snRNA-seq data. We repeated this reference with and without the DEGs (i.e., new models trained with less gene features) as computed above.

Comparison of snRNA-seq transformed cell types with real scRNA-seq cells

For each cell type in each dataset (Figure 1B) we compared the expression profile obtained after every snRNA transformation with the corresponding profile from the scRNA-seq reference. For both modalities, we randomly sampled 50 cells per cell type and aggregated them to reduce sparsity by summing counts to form a pseudobulk vector; the minimum number of cells across all cell types and datasets. We normalized the expression to counts per million and log-transformed to makes samples comparable across library sizes, and log makes the similarity reflect relative expression patterns rather than being dominated by highly expressed genes. By using raw counts, the cosine similarity would be dominated by total read depth, not true biological differences. We then calculated cosine similarity between each transformed snRNA-seq vector and its scRNA-seq counterpart. The procedure was repeated for all transformations, with untransformed snRNA-seq serving as a negative control and scRNA-seq as the positive control. The similarity scores were then summarized across cell types.

Comparison of transformed cell types with real bulks

We created pseudobulks as outlined in the previous Pseudobulks section (same number of cells and sampling logic). We created 100 realistic-proportioned pseudobulks and 100 random-proportioned pseudobulks for each reference type (PCA·LS, scVI·LS, scVI-cond, and repeated without -DEGs). For all pseudobulks, we used all scRNA-seq cells and added the snRNA-seq "missing" cells (adipocytes and neutrophils) either raw or with each of our transformations listed above. We aimed to see which transformation made the pseudobulks containing the transformed cells be closer to the real bulks by computing the cosine similarity. We first library-normalized the data to counts per million to remove the sequencing depth (or cell number) variation, and log + 1 the data to stabilize the variance. We then computed the cosine similarities. For each transform, we obtained a distribution of cosine similarity scores: one value for every pseudobulk x realbulk comparison. We then computed the 95% bootstrapped CI of the mean with 1000 iterations.

Deconvolution results and comparison

For deconvolution, we used BayesPrism[10] methodology through InstaPrism,[47] a probabilistic framework that leverages the expression profiles of the cell reference to deconvolve bulk mixtures that has been shown to outperform others in previous work.[28] We executed the InstaPrism deconvolution for 5000 iterations for each bulk, per reference dataset, including the controls using only the scRNA-seq data ("scRNA All (PosCtrl)") and only the snRNA-seq data ("snRNA All (NegCtrl)"), as well as the transformed hybrids. The deconvolution output consisted of estimated cell type proportions for each bulk/pseudobulk sample.

In the case of the simulation experiments (i.e., pseudobulks) we have ground truth proportions which we can use to compute performance metrics. We compared the estimated cell-type proportions with the known ground-truth proportions with two quantitative metrics: Pearson correlation to assess the linear concordance between predicted and true proportions, and RMSE to quantify the overall estimation error.

We evaluated performance under three scenarios for each bulk:

- **All Cells:** The evaluation was performed using the entire set of cell types present in the simulated bulk (all proportions of all cells estimated).
- **Non-Removed Cells:** The analysis was repeated after excluding the held-out cell type from the evaluation. This scenario represents the case where the reference already contains the cell types that are present in the bulk.
- **Removed Cell Only:** In a realistic missing-cell scenario, one cell type was intentionally removed from the scRNA-seq reference and replaced with the transformed snRNA-seq data. Performance for this held-out cell type was evaluated separately. For this scenario, controls (i.e., references that did not have any held out any cell type) were excluded since there is no direct comparison (no held-out cell type).

For the simulations, we show the mean performance per transforms along with the 95% bootstrapped CI of the mean with 1000 iterations. For the non-simulation experiments (real bulks deconvolved with each transform type, and with each patient's datasets), we do not have ground truth proportions, so we evaluate the robustness as outlined below (see Real adipose bulks deconvolution robustness).

For the comparison of using scRNA-seq vs. snRNA-seq as reference in pseudobulks made from scRNA-seq cells, we also conducted independent two-sample Student's t-test to compare the mean performance metrics between scRNA-seq reference and snRNA-seq reference across each of the datasets. We tested for differences in the Pearson correlation coefficients and the RMSE deconvolution performance values by performing two-tailed t-tests (assuming equal variances), with any missing values omitted on a pairwise basis. Statistical significance was evaluated at the 0.005 level.

Deconvolution with DWLS and SCDC

We also ran deconvolution of the exact same references as used described above for BayesPrism but using the SCDC[49] and Scaden[48] methods through omnideconv[32] (version 1). We used the default omnideconv parameters for Scaden and SCDC in all experiments. We trained a different model for Scaden for each reference to ensure no cell-type information would be used between references or models. SCDC can leverage multiple batch information in the reference, but in order to test the effect of our transformations rather than the batch weighting of SCDC, every reference was treated as one batch.

Gene ontology analysis of intersection genes

We identified a list of genes that is differentially expressed in at least one cell type across all 3 human datasets (ADP, PBMC, MBC). We used GOrilla (Gene Ontology enRIchment anaLysis and visuaLizAtion tool)[46] to test for over-representation of GO Cellular Component terms in the intersection gene set. The "target" list comprised the "intersection genes", and the "background" list comprised every gene detectable in all three of the datasets (ADP, PBMC, MBC). Enrichment was calculated with the two-list (target vs. background) hypergeometric test implemented in GOrilla, and p-values < 0.05 were considered significant. We considered the first 30 components for visualization in Figure S3.

Real adipose bulks data and single modality data preprocessing

Bulk RNA-seq data from 331 subcutaneous adipose tissue samples (METSIM cohort, GEO GSE135134[20]) were processed by first importing TPM expression values from the publicly available downloaded file (see Data Availability). Gene-level annotation was performed using a Gencode v47 basic GTF file.[55] Finally, only the genes common to the bulk data and corresponding snRNA-seq and scRNA-seq datasets were retained, and the resulting expression matrix was saved for downstream analyses.

For the accompanying scRNA-seq and snRNA-seq datasets, we used the same data source[20] (not same datasets) as used for the adipose data in the deconvolution experiment. For this experiment, we filtered to only datasets that are the same tissue type as the real bulks (subcutaneous adipose tissue) that were not used as references or for deconvolution in the previous experiment. This yielded 7 scRNA-seq and 12 snRNA-seq patient datasets. We filtered each of the datasets independently. We added a table with all data links and processing parameters for each dataset to the GitHub repository and Zenodo (/data/details/Data_Details.xlsx).[33,34]

Real bulks deconvolution robustness (per transform and per patient)

We used deconvolution, as described above, to estimate the proportions of cell types in 434 real bulk samples. We used the adipose tissue datasets as references (7 scRNA-seq datasets, 11 snRNA-seq datasets). These datasets had two additional cell types in snRNA-seq that were not present in any scRNA-seq dataset (fat cells and neutrophils), and one cell in scRNA-seq not present in snRNA-seq, making them an ideal dataset to test what happens in a true research setting. We created a reference with all scRNA-seq cells, and the two additional cells from snRNA-seq, either raw as a control or transformed with each of our transforms (described above).

Previous work has described robustness (i.e., consistency in predictions) as a measure of performance[28] in deconvolution. We did two experiments to evaluate the performance of our transformations; we compared the predicted proportions of each transformation to the predicted proportions of the other transformations. We also tested the robustness of our transformations by comparing the predicted proportions if we only use fat cells from one patient at a time (i.e., inter patient robustness). We created references with all scRNA-seq cell types from all patients, snRNA-seq neutrophils from all patients (these cells have low quantities in all patients, so we pooled them), and snRNA-seq fat cells from each patient at a time transformed with each of our transforms.

We computed the cosine similarity between each transformation's predicted proportions as two long vectors (transform to transform robustness), and we computed the cosine similarity between each patient's predicted proportions per transform (e.g., patient 1 cells PCA·LS transform vs. patient 2 cells PCA·LS transform). Finally, we compare the cosine similarities for each transform, both across transformations and across patients. We plot each in x and y axes respectively (Figure 3B).

Composite score for each transform

We used the RMSE and Pearson correlation values from the simulation experiments (described above), along with the robustness/ consistency measures per patient and per transform (described above) to get a new composite score per transform.

For each simulation dataset (ADP, PBMC, MSB, MBC), we took every held-out-cell evaluation (per-sample Pearson correlation and RMSE) and normalized each metric to the range [0,1] using min-max scaling, applied separately within each dataset. For RMSE, we first inverted the values by subtracting them from the dataset maximum (equivalent to multiplying by -1 before scaling), so that higher values indicate better accuracy. We then averaged the normalized Pearson and RMSE values across all samples belonging to the same transform within that dataset. Next, we collapsed across datasets by taking the mean of each transform's per-dataset normalized Pearson and normalized RMSE. Finally, we defined a single "accuracy" score for each transform as the arithmetic mean of its two normalized metrics. This yields one score that summarizes the RMSE and Pearson correlation across all held-out cell types.

For each reference transformation, we quantified its consistency along two orthogonal axes (across donors and across transforms) and then combined them into a single "consistency score." First, from the pair-wise donor-to-donor similarity table (all cosine similarities between every two donors' predicted proportion vectors for a given transform), we min-max scaled those cosine values to [0, 1] and averaged them to yield a per-donor score for each transform. Second, from a transform-to-transform cosine-similarity matrix (excluding self-comparisons), we likewise flattened the off-diagonal entries into a long list, applied the same min-max scaling, and averaged per transform to obtain a per-transform score. Finally, we defined the overall "robustness score" as the simple mean of the per-donor score and per-transform score, thereby giving equal weight to between-donor and between-reference agreement. This yields one score that summarizes the cosine similarities across transforms and donors. This gave us two normalized values per transform; one that shows the accuracy seen in pseudobulk experiment (Figure 3E, y-axis), and one that shows the robustness in real data (Figure 3E, x-axis).

QUANTIFICATION AND STATISTICAL ANALYSIS

All quantitative analyses were performed using Python (version 3.9) and R (version 4.2). Performance of bulk RNA-seq deconvolution was evaluated using Pearson correlation and root mean squared error (RMSE) between estimated and ground-truth cell-type proportions in simulation experiments. Pearson correlation quantified linear concordance across cell types, while RMSE quantified absolute estimation error. In all simulation analyses, n represents the number of pseudobulk samples evaluated per reference transformation and dataset.

Bootstrapped 95% confidence intervals (CIs) of the mean were computed using 1,000 bootstrap resamples unless otherwise stated. For comparisons between scRNA-seq and snRNA-seq reference performance, two-tailed independent Student's t-tests were performed separately for Pearson correlation and RMSE within each dataset, assuming equal variances, with statistical significance assessed at $p < 0.005$. Missing values were omitted on a pairwise basis.

For experiments involving real bulk RNA-seq data without ground-truth proportions, robustness was assessed using cosine similarity between predicted cell-type proportion vectors. Two forms of robustness were evaluated: inter-transform robustness, defined as cosine similarity between predictions obtained using different reference transformations for the same bulk samples, and inter-patient robustness, defined as cosine similarity between predictions obtained using references constructed from different donors for the same transformation. Cosine similarity values were summarized using mean and bootstrapped 95% CIs.

Composite accuracy and robustness scores were computed by min-max normalizing individual metrics to the range [0,1] within each dataset. RMSE values were inverted prior to normalization so that higher values indicate better performance. Accuracy scores were defined as the mean of normalized Pearson correlation and normalized RMSE, while robustness scores were defined as the mean of normalized inter-transform and inter-patient cosine similarity measures. All normalization and aggregation steps were performed independently for each dataset before averaging across datasets. Statistical analyses and plotting were performed using NumPy, SciPy, pandas, and matplotlib.

Cell Reports Methods, Volume *6*

Supplemental information

Integrating single-cell and single-nucleus datasets improves bulk RNA-seq deconvolution

Adriana Ivich and Casey S. Greene

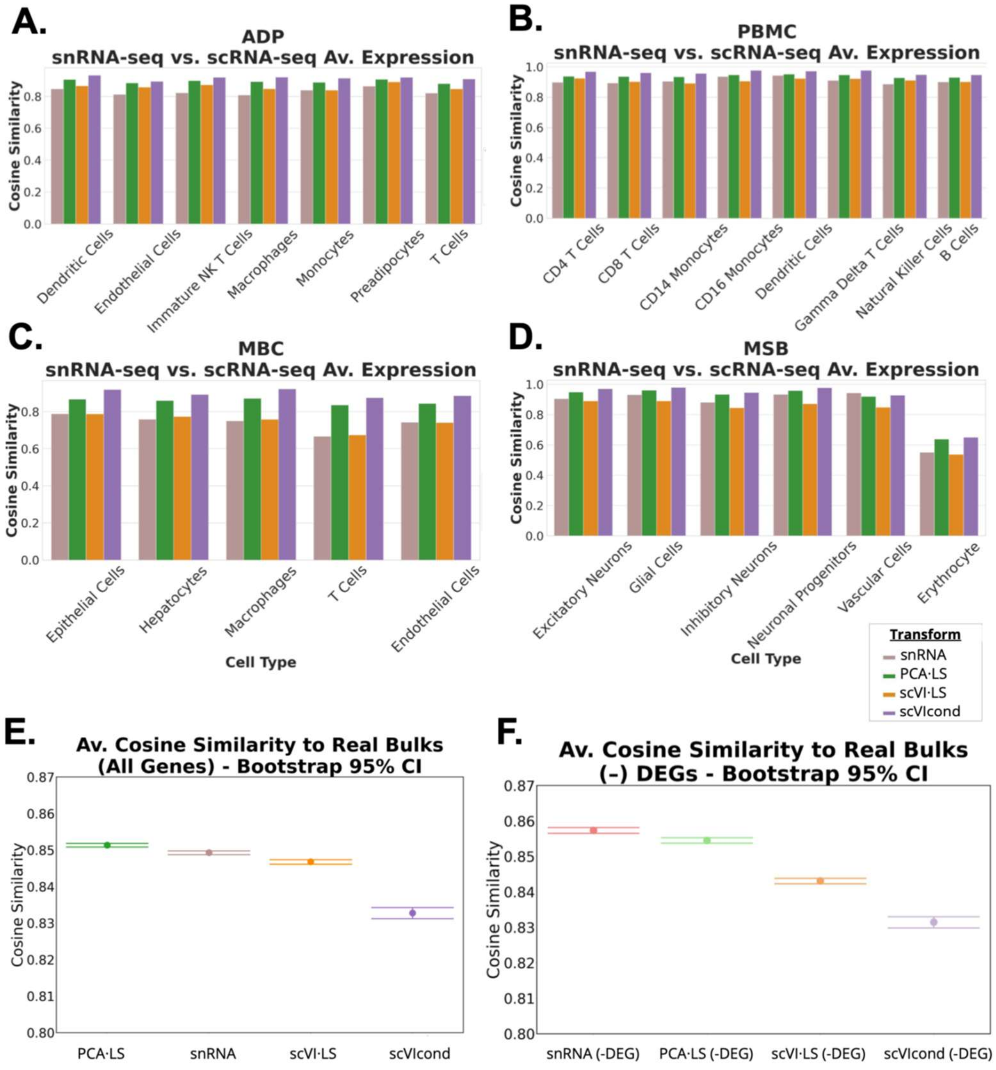

Figure S1. ScVIcond improves cell-type alignment, whereas PCA·LS-transformed snRNA-seq best matches bulk expression, related to Figures 2–4.

Comparison of transformed snRNA-seq data with matched scRNA-seq profiles and with bulk RNA-seq. (A–D) For each dataset (A: ADP, B: PBMC, C: MBC, D: MSB), we computed the cosine similarity between the mean CPM-normalized, log-transformed expression vector for each scRNA-seq cell type and the corresponding snRNA-seq cell type after transformation (PCA·LS, scVI·LS, scVIcond) or without transformation (raw snRNA-seq). Across datasets and cell types, transformations generally increase similarity relative to raw snRNA-seq; scVIcond shows the highest cell-type alignment overall with PCA·LS typically close behind, whereas scVI·LS is often comparable to raw snRNA-seq and can be lower for some MSB cell types. Cosine similarity captures the agreement in relative expression patterns (vector "shape") independent of overall amplitude (1 = identical patterns). (E) Adipose pseudobulks were created by aggregating scRNA-seq cell types and adding two snRNA-only cell types (adipocytes and neutrophils) either as raw snRNA-seq, transformed snRNA-seq, or using all snRNA-seq as a control; each pseudobulk was compared to 434 bulk RNA-seq profiles using cosine similarity in gene space. In this bulk comparison, pseudobulks with PCA·LS-transformed additions show higher similarity than pseudobulks with raw snRNA-seq additions, whereas scVIcond yields the lowest similarity. (F) Repeat of E using a different feature space in which genes differentially expressed between matched scRNA-seq and snRNA-seq cell types were removed (-DEG; see STAR Methods for DEG definitions). In the -DEG setting, DEG removal alone yields the highest bulk similarity, followed by PCA·LS−DEG. Dots indicate mean cosine similarity; bars show bootstrapped 95% confidence intervals; y-axes are truncated to highlight variation.

scRNA, single-cell RNA-seq; snRNA, single-nucleus RNA-seq; ADP, adipose tissue; PBMC, peripheral blood mononuclear cells; MBC, metastatic breast cancer; MSB, mouse E18 brain.

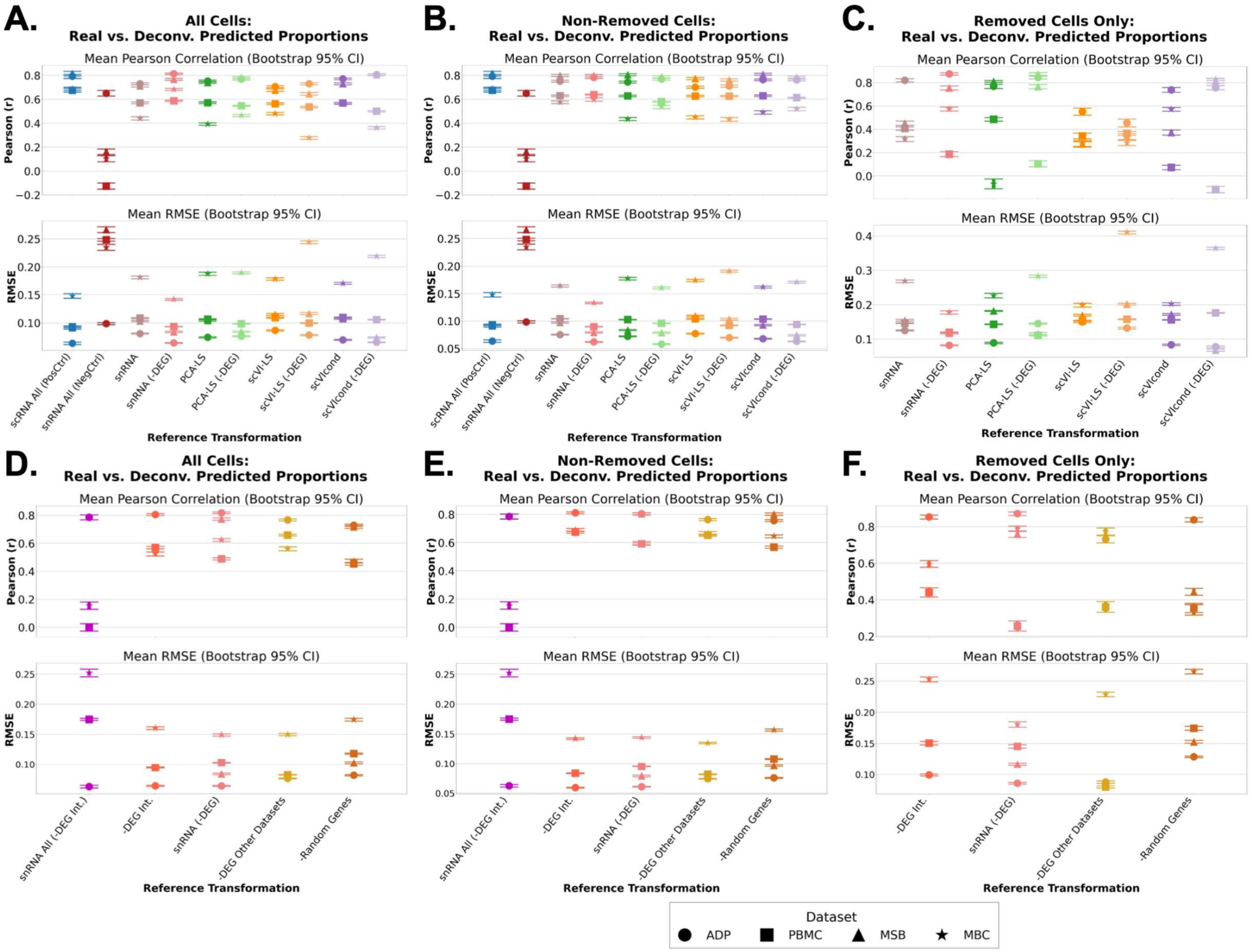

Figure S2. Pseudobulk deconvolution accuracy with each cell type in scRNA-seq held out and transformed, **related to Figure 2.**

Each plot shows the Pearson correlation value (top panels) and the RMSE values (bottom panel) for the ground truth pseudobulk (simulated) proportions and the predicted proportions. We hold out one cell type at a time from each dataset and replace that cell type's expression with a snRNA-seq equivalent with each of the transformations or controls (scRNA-seq All and snRNA-seq All) on the x axis of each plot. Each dot represents the mean metric (r or RMSE) across datasets (Figure 1B) and the bars represent the 95% bootstrapped confidence interval of the mean. We evaluated 3 scenarios (see Methods for details). (A and D) All cells included in performance metrics calculations. (D and E) Non-Removed cells only included in performance metrics calculations. (C and F) Only removed cells included in performance metrics calculations. Y-axes are truncated to highlight the variation. Combined metrics for all datasets are shown in Figure 2.

scRNA-seq: single-cell RNA-seq, RMSE: root mean squared error, snRNA-seq: single-nucleus RNA-seq.

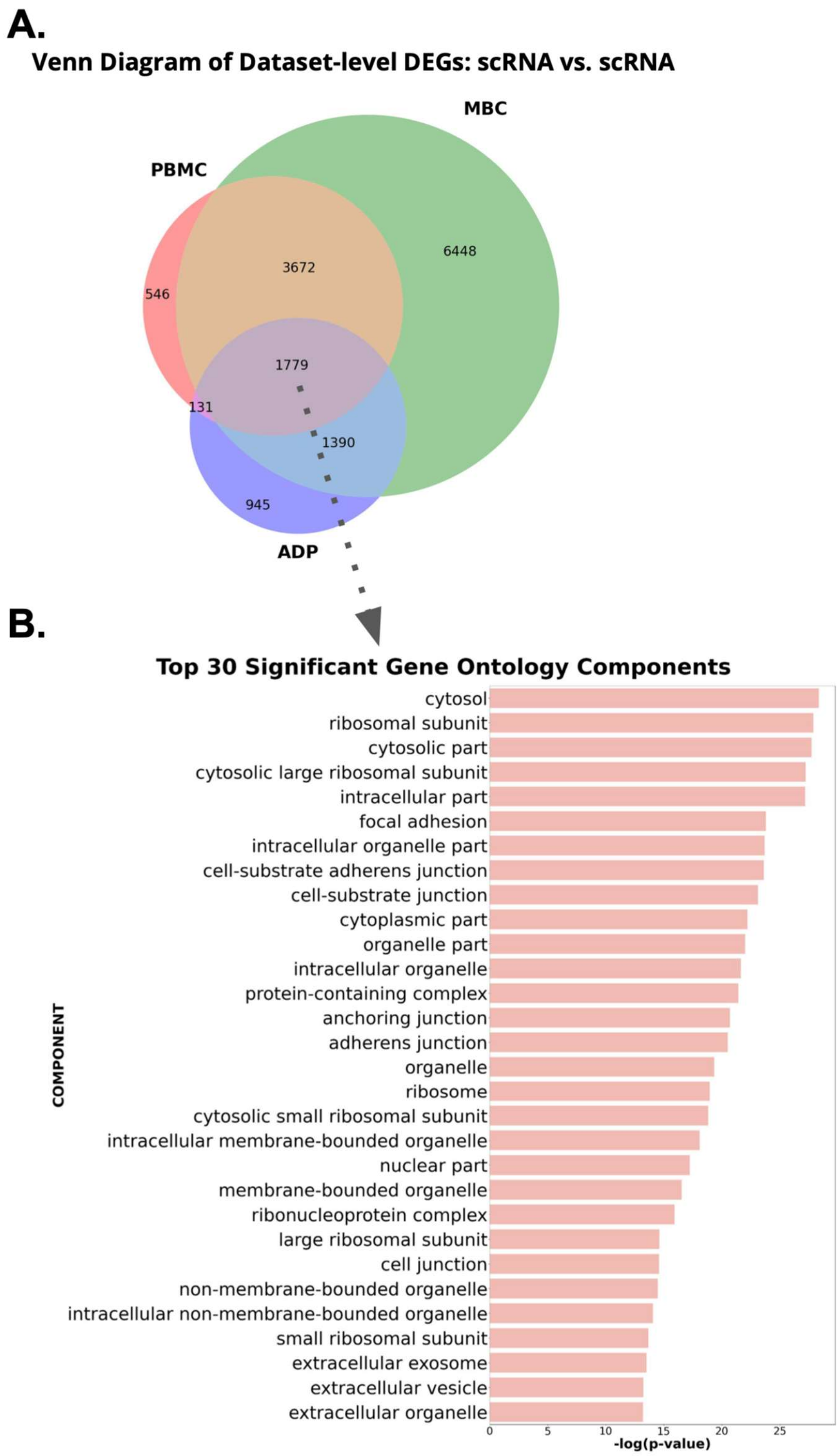

Figure S3. Shared and unique scRNA-seq vs snRNA-seq differentially expressed genes across three human datasets, with GO-component enrichment of the common signature, related to Figures 2-4 and STAR Methods.

DEGs between scRNA-seq and snRNA-seq cells of the same cell types in the three human datasets. a. Venn diagram showing the number of DEGs in common and distinct between cell types. b. Significant (GOrilla default of p <0.005) gene ontology terms (component) identified from the intersection genes (target) compared to the full gene list (all genes in common between datasets).

DEG: differentially expressed gene. ScRNA-seq: single-cell RNA-seq. snRNA-sequencing: single-nucleus RNA-sequencing. ADP: adipose tissue. PBMC: peripheral blood mononuclear cells. MBC: metastatic breast cancer.

Differential Gene Expression per Dataset and Cell Type (snRNA-seq vs. scRNA-seq)

Cell Type	Number of DEGs	Percentage of Total
**Peripheral Blood Mononuclear Cells (PBMC) Dataset**		
Natural Killer Cells	1915	8.73%
Gamma Delta T Cells	2327	10.61%
Dendritic Cells	1429	6.51%
CD4 T Cells	4009	18.28%
CD8 T Cells	3531	16.10%
B Cells	3233	14.74%
CD16 Monocytes	3785	9.61%
CD14 Monocytes	3233	17.26%
**All Cell Types' Union**	**6128**	**27.94%**
**All Cell Types' Intersection**	**710**	**3.24%**
**Adipose Tissue (ADP) Dataset**		
Preadipocytes	3556	12.64%
T Cells	520	1.85%
Immature NK T Cells	336	1.19%
Macrophages	1403	4.99%
**All Cell Types' Union**	**4245**	**15.09%**
**All Cell Types' Intersection**	**186**	**0.66%**
**Metastatic Breast Cancer (MBC) Dataset**		
Endothelial Cells	5422	17.88%
T Cells	8738	28.82%
Immature NK T Cells	8546	28.19%
Macrophages	10081	33.25%
**All Cell Types' Union**	**13289**	**43.83%**
**All Cell Types' Intersection**	**3576**	**11.80%**
**Mouse E18 Brain (MSB) Dataset**		
Inhibitory Neurons	5069	19.61%
Neuronal Progenitors	4299	16.63%
Glial Cells	4455	17.24%
Excitatory Neurons	5648	21.85%%
**All Cell Types' Union**	**7289**	**28.20%**
**All Cell Types' Intersection**	**2917**	**11.29%**
**Common Cells Between Datasets**		
Macrophages (ADP and MBC) **Union**	10531	3.9%
Macrophages (ADP and MBC) **Intersection**	953	4.0% ADP, 66.7% MBC
T Cells (ADP and MBC) **Union**	8911	9.06%
T Cells (ADP and MBC) **Intersection**	347	9.45% ADP, 67.9% MBC

Table S1. Number of union and intersection genes per cell type in each of the datasets, related to Figures 2-4. First row (left) shows the cell type, middle row shows the number of DEGs between that cell type in scRNA-seq vs. scRNA-seq. The right-most row shoes the percentage of total genes that are classified as DEGs. For each dataset, we note the number of genes that are in common between the cell types, both union (all) and intersection (common between all cell types). The human datasets contained 2 cell types in common across datasets, and we note the union and intersection of the DEGs between datasets as well.
DEG: differentially expressed gene. scRNA-seq: single-cell RNA-seq. snRNA-seq: single-nucleus RNA-seq.
